# New α-Methylene-γ-Butyrolactone Derivatives as Potential Fungicidal Agents: Design, Synthesis and Antifungal Activities

**DOI:** 10.3390/molecules21020130

**Published:** 2016-01-22

**Authors:** Yongling Wu, Delong Wang, Yanqing Gao, Juntao Feng, Xing Zhang

**Affiliations:** Research & Development Center of Biorational Pesticide, Key Laboratory of Plant Protection Resources and Pest Management of Ministry of Education, Northwest A & F University, Xinong Road 22, Yangling 712100, Shaanxi, China; wuyongling39@126.com (Y.W.); rizhaoalong@163.com (D.W.); gaoyanqinggc@nwsuaf.edu.cn (Y.G.); zhxing1952@126.com (X.Z.)

**Keywords:** α-methylene-γ-butyrolactone, ester and ether derivatives, antifungal activity, quantitative structure-activity relationships (QSAR), heuristic method

## Abstract

In consideration of the fact that the α-methylene-γ-butyrolactone moiety is a major bio-functional group in the structure of carabrone and possesses some agricultural biological activity, forty-six new ester and six new ether derivatives containing α-methylene-γ-butyrolactone moieties were synthesized, and their fungicidal activities against *Colletotrichum lagenarium* and *Botrytis cinerea* were investigated. Most of the synthesized compounds showed moderate to significant fungicidal activity. Among them, halogen atom-containing derivatives showed better activity than others, especially compounds **6a**,**d** which exhibited excellent fungicidal activity against *C. lagenarium*, with IC_50_ values of 7.68 and 8.17 μM. The structure-activity relationship (SAR) analysis indicated that ester derivatives with electron-withdrawing groups on the benzene ring showed better fungicidal activity than those with electron-donating groups. A quantitative structure-activity relationship (QSAR) model (*R*^2^ = 0.9824, *F* = 203.01, *S*^2^ = 0.0083) was obtained through the heuristic method. The built model revealed a strong correlation of fungicidal activity against *C. lagenarium* with the molecular structures of these compounds. These results are expected to prove helpful in the design and exploration of low toxicity and high efficiency α-methylene-γ-butyrolactone-based fungicides.

## 1. Introduction

Plant pathogenic fungi remain a main cause of plant diseases, which can infect any tissue of a plant and cause severe yield agricultural product losses [1,2,3]. Moreover, the presence of some phyto-fungal mycotoxins can be harmful to animal and human health [4]. *Colletotrichum lagenarium* and *Botrytis cinerea* are the most common plant pathogenic fungi, and can cause cross-infections between diseased and healthy plants [5,6,7]. In addition, they cause significant reductions of crop yield and quality [8]. Traditional chemical fungicides play an important role in killing or controlling target fungi directly, but sometimes cause adverse effects to the environment and food, and often create fungicide resistance [9]. Therefore, it is urgent to develop novel and effective fungicidal agents to protect plants.

**Figure 1:**
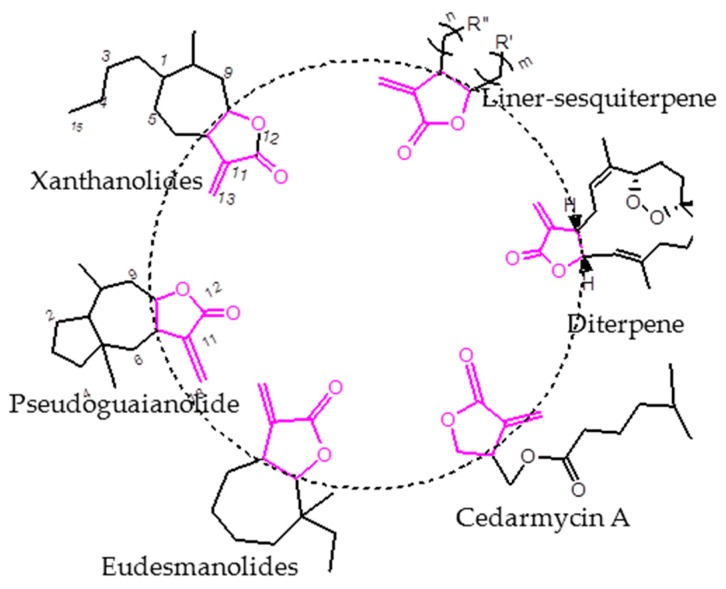
Some representative sesquiterpenoid structures.

The α-methylene-γ-butyrolactone ring can be found as a key substructural unit in many sesquiterpenoids (Figure 1). It exhibits multiple biological properties, including antibacterial, cytotoxic, antiinflammatory, antioxidant, allergenic and antimicrobial activity [10,11,12,13,14,15,16]. In our previous research, we found that carabrone (which is isolated from fruits of *Carpesium macrocephalum*) and its derivatives exhibited potent antifungal activity against *C. lagenarium*, and the structure-activity relationship (SAR) analysis of these compounds indicated that the α-methylene-γ-butyrolactone ring was a major biofunctional group in the carabrone structure [17,18,19,20]. Besides, γ-monosubstituted compounds of the α-methylene-γ-lactone ring have also been synthesized, and we concluded that aromatic substituents directly fused to the γ-position improved the potency more effectively than alkyl groups. Meanwhile, the cytotoxicity was tested to ensure the selectivity of the fungicidal effects [21].

It is virtually and economically impossible to develop and screen candidates with fungicidal activity from among numberless compounds. The development of new quantitative structure-activity relationship (QSAR) methods, with simple molecular indexes, is a promising shortcut to resolve the cost and time issues [22]. The QSAR method enables the calculation of numerous quantitative descriptors on the basis of molecular structural information and is very useful to optimize important aspects such as fungicidal activity or toxicity. Meanwhile, QSAR is useful in provide further guidance for the design and development of potential new fungicides [23,24].

In order to obtain novel natural product-based fungicides, two series of derivatives based on γ-monosubstituted α-methylene-γ-butyrolactone rings were synthesized on the basis of their molecular similarity. The fungicidal activities of these compounds against *C. lagenarium* and *B. cinerea* were investigated and their structures were characterized by ^1^H-NMR, ^13^C-NMR, and HRMS spectrometric analysis. Meanwhile, the cytotoxicity was tested to ensure selectivity of the antifungal effects. Moreover, a QSAR study was also performed on all of the derivatives using the Gaussian and CODESSA software packages, which can correlate their structural features with their fungicidal activity.

## 2. Results and Discussion

### 2.1. Synthesis

**Scheme 1:**
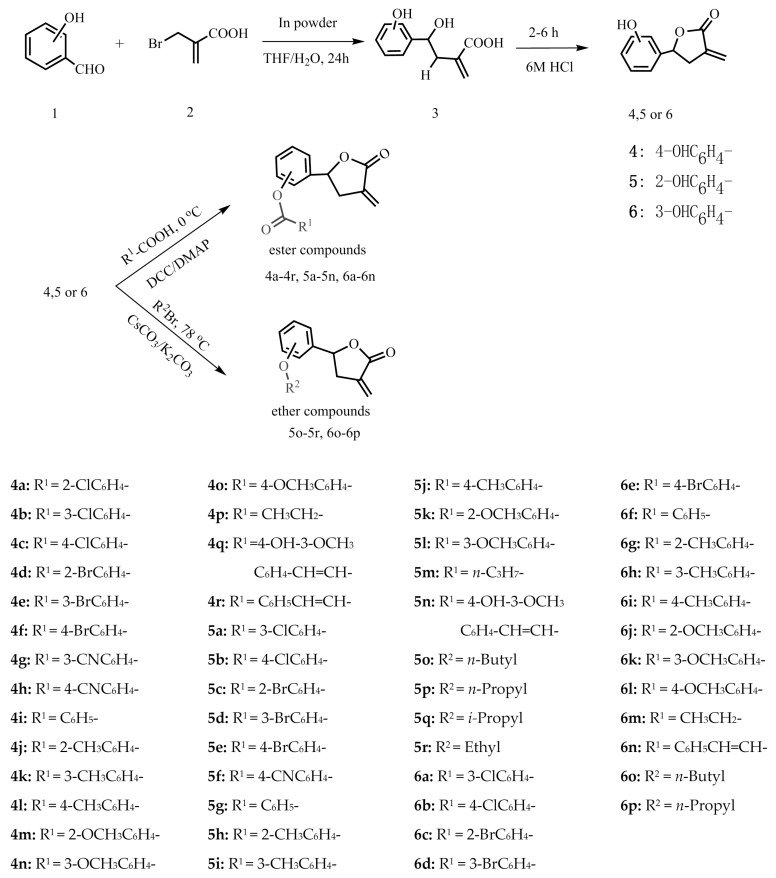
Synthetic route of the title compounds.

Three kinds of intermediate compounds **4**–**6** were prepared by the cyclization of γ-hydroxy-α-methylene esters, which were obtained under mild aqueous reaction conditions through indium-mediated Barbier allyl addition to aldehydes [25]. In order to investigate the structure-activity relationships, different acids were reacted with the three kinds of intermediate compounds to obtain the corresponding ester compounds. Then, six new ether compounds were obtained by reacting with them with brominated alkanes. The structures of all the derivatives were characterized by ^1^H-NMR, ^13^C-NMR and high-resolution electrospray ionization mass spectrometry (HR-ESI-MS). The synthetic routes are shown in Scheme 1.

### 2.2. Fungicidal Activity and Structure-Activity Relationships (SAR)

#### 2.2.1. Fungicidal Activity of the Title Compounds against *C. lagenarium*

The results of the fungicidal activity against *C. lagenarium* are summarized in Table 1, from which it can be seen that the halogen atom-containing derivatives exhibited significant fungicidal activity against this species. The following three main SARs were obtained: first, the introduction of the electron-withdrawing groups Cl, Br, and CN onto the benzene ring dramatically increased the potency. Compounds **4a–f**, **5a–e**, and **6a–e** (IC_50_ < 18 μM) exhibited fungicidal activity approximately ten to twenty fold higher than the intermediate compounds **4–6**, respectively. It was notable that the IC_50_ values of **6a**,**d** were approximately two fold lower than those of chlorothalonil, a commercial fungicide. Meanwhile, the electron-donating groups CH_3_ and CH_3_O introduced onto the benzene ring to give **4j–o**, **5h–l** and **6g–l** (IC_50_ > 126 μM) greatly weakened the potency, which was similar to that of the fatty acid derivatives **4p**, **5m** and **6m**. It can be concluded that the electronic effect of the substituent on the benzene ring is important for the fungicidal activity of α-methylene-γ-butyrolactone groups. Second, intermediate compound **6** was found to have higher activity than the corresponding intermediate compounds **4** and **5**. Meanwhile, *meta*-substitution on the benzene ring (compounds **6a–p**) was found to improve the potency significantly compared with the corresponding *ortho*- and *para*-substitution patterns (compounds **4a–r** and **5a–r**). This result suggests that the steric effect should be considered and substitution patterns on the benzene ring have an important influence on the fungicidal activity. Third, all of the synthesized ether compounds exhibited lower fungicidal activity against *C. lagenarium* than the corresponding ester compounds. It was notable that the cinnamic acid and fumalic acid derivatives **4q–r**, **5n** and **6n** containing an unsaturated bond showed higher fungicidal activity against *C. lagenarium*.

**Table 1:** *In vitro* fungicidal activity of compounds against *C. lagenarium* and *B. cinerea*.

Compd.	*C. lagenarium*		*B. cinerea*	Compd.	*C. lagenarium*		*B. cinerea*
	IC_50_^a^, μM	pIC_50_	IC_50_^a^, μM		IC_50_^a^, μM	pIC_50_	IC_50_^a^, μM
**4a**	13.96	−1.145	29.81	**5k**	291.95	−2.465	306.85
**4b**	8.99	−0.954	22.13	**5l**	283.99	−2.453	303.36
**4c**	12.74	−1.105	27.31	**5m**	173.74	−2.240	205.50
**4d**	15.62	−1.194	24.09	**5n**	10.27	−1.012	20.57
**4e**	8.76	−0.943	30.24	**5o**	413.72	−2.617	456.91
**4f**	14.38	−1.158	23.01	**5p**	499.33	−2.698	524.54
**4g**	52.87	−1.723	60.61	**5q**	428.93	−2.632	446.08
**4h**	65.63	−1.817	77.00	**5r**	519.42	−2.716	548.63
**4i**	95.38	−1.979	111.77	**6a**	7.68	−0.885	23.32
**4j**	192.44	−2.284	198.67	**6b**	9.24	−0.966	29.08
**4k**	177.96	−2.250	193.28	**6c**	10.27	−1.012	27.45
**4l**	188.93	−2.276	209.12	**6d**	8.17	−0.912	25.99
**4m**	238.97	−2.378	243.35	**6e**	9.70	−0.987	27.15
**4n**	212.50	−2.327	224.16	**6f**	73.14	−1.864	101.94
**4o**	219.44	−2.341	230.85	**6g**	159.33	−2.202	179.62
**4p**	206.51	−2.315	236.62	**6h**	125.71	−2.099	147.84
**4q**	8.93	−0.951	21.50	**6i**	136.55	−2.135	163.84
**4r**	44.51	−1.648	56.32	**6j**	162.30	−2.210	196.45
**5a**	16.77	−1.225	30.57	**6k**	135.95	−2.133	161.43
**5b**	17.74	−1.249	34.51	**6l**	154.27	−2.188	179.33
**5c**	15.94	−1.202	34.60	**6m**	173.23	−2.239	202.97
**5d**	13.41	−1.127	22.93	**6n**	24.25	−1.385	40.26
**5e**	15.35	−1.186	36.10	**6o**	242.03	−2.384	280.01
**5f**	79.07	−1.898	95.02	**6p**	266.25	−2.425	298.26
**5g**	170.80	−2.232	193.75	**4**	160.63	−2.206	207.99
**5h**	215.94	−2.334	229.37	**5**	238.61	−2.378	279.96
**5i**	181.69	−2.259	193.87	**6**	117.12	−2.069	139.85
**5j**	207.63	−2.317	259.27	**Chlorothalonil ^b^**	4.21	−0.624	8.31

Note: ^a^ All 50% inhibition concentration (IC_50_) values are presented as the means ± SD (*n* = 3), μM; ^b^ Commercial fungicide, chlorothalonil was used as the positive control.

#### 2.2.2. Fungicidal Activity of the Title Compounds against *B. cinerea*

The results of the fungicidal activity against *B. cinerea* are summarized in Table 1, from which we can see that compounds **4a**–**f**, **4q**, **5a**–**e**,**n** and **6a**–**e** exhibited moderate fungicidal activity against *B. cinerea*. All of the test compounds were less effective than against *C. lagenarium*.

### 2.3. QSAR Study on the Fungicidal Activity against C. lagenarium

In general, descriptors used in QSAR can be categorized as constitutional, topological, geometrical, electrostatic, quantum chemical, and thermodynamic. There are many regression approaches available for the CODESSA 2.7.15 software, such as the best multi-linear, multi-linear regression, principal component analysis, partial least square regression, and heuristic regression [26]. In view of the number of samples and descriptors used in this study, the heuristic regression was selected for developing the QSAR model.

**Figure 2:**
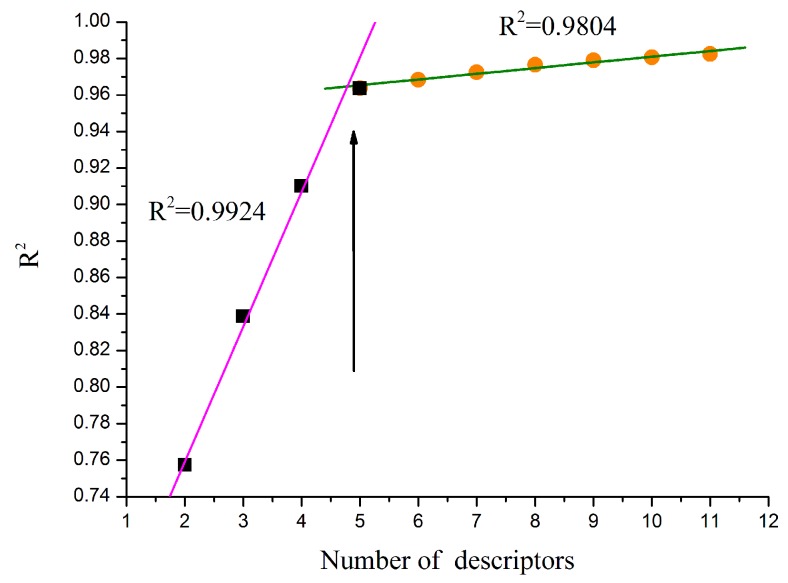
The “breaking point” rule results.

Determining the number of descriptors is an important step. The “breaking point” rule was used in the improvement of the statistical quality of the model, as described in Figure 2, the *R*^2^ value of the heuristic regression had a dramatic increase before the number of the descriptors reached 5, descriptors with high *t* values were accepted and those with low *t* values were rejected. After the number of the descriptors reached a certain value, the improvement of the regression model became less insignificant (Δ*R*^2^ < 0.02–0.04) [27]. In addition, the number of the descriptors complies to the linear regressions given by Equation (1):

*N* ≥ 3 (*K* + 1) (1)

where *N* is the number of sample compounds and *K* is the number of descriptors. Thus, the final model with five descriptors was selected as the best model. The values of the five descriptors of compounds can be found in Table 2.

**Table 2:** Fungicidal activity and structural descriptors of the title compounds.

No.	Compd.	pIC_50_	Structural Descriptors				
			*N*_o_	*q*^C^_max_	MAOEP	*q*^H^_max_	*q*^H^_min_
1	**4a**	−1.1450	1.6111	0.3570	1.9821	0.1606	0.1084
2	**4b**	−0.9540	1.6111	0.3568	1.9820	0.1716	0.1084
3	**4c**	−1.1050	1.6111	0.3571	1.9820	0.1628	0.1084
4	**4d**	−1.1940	1.6111	0.3532	1.9751	0.1613	0.1078
5	**4e**	−0.9430	1.6111	0.3571	1.9851	0.1712	0.1084
6	**4f**	−1.1580	1.6111	0.3555	1.9669	0.1631	0.1085
7	**4g**	−1.7230	1.5946	0.3558	1.9135	0.1708	0.1088
8	**4h**	−1.8170	1.5946	0.3533	1.9134	0.1655	0.1088
9	**4i**	−1.9790	1.5278	0.3575	1.9133	0.1603	0.1082
10	**4j**	−2.2840	1.4872	0.3595	1.9132	0.1601	0.0821
11	**4k**	−2.2500	1.4872	0.3577	1.9133	0.1602	0.0872
12	**4l**	−2.2760	1.4872	0.3588	1.9133	0.1601	0.0885
13	**4m**	−2.3780	1.5250	0.3658	1.9138	0.1598	0.0751
14	**4n**	−2.3270	1.5250	0.3558	1.9133	0.1721	0.0735
15	**4o**	−2.3410	1.5250	0.3616	1.9135	0.1608	0.0771
16	**4p**	−2.3150	1.4688	0.3342	1.9125	0.1610	0.0843
17	**4q**	−0.9210	1.5333	0.3441	1.9135	0.2228	0.0668
18	**4r**	−1.6480	1.5000	0.3435	1.9135	0.1607	0.1074
19	**5a**	−1.2250	1.6111	0.3597	1.9820	0.1635	0.1077
20	**5b**	−1.2490	1.6111	0.3598	1.9820	0.1647	0.1078
21	**5c**	−1.2020	1.6111	0.3514	1.9838	0.1594	0.1080
22	**5d**	−1.1270	1.6111	0.3596	1.9667	0.1736	0.1079
23	**5e**	−1.1860	1.6111	0.3581	1.9788	0.1649	0.1081
24	**5f**	−1.8980	1.5946	0.3563	1.9132	0.1670	0.1092
25	**5g**	−2.2320	1.5278	0.3602	1.9131	0.1590	0.1068
26	**5h**	−2.3340	1.4872	0.3643	1.9126	0.1546	0.0871
27	**5i**	−2.2590	1.4872	0.3603	1.9131	0.1587	0.0848
28	**5j**	−2.3170	1.4872	0.3614	1.9131	0.1585	0.0859
29	**5k**	−2.4650	1.5250	0.3741	1.9121	0.1568	0.0710
30	**5l**	−2.4530	1.5250	0.3593	1.9131	0.1752	0.0718
31	**5m**	−2.2400	1.4286	0.3432	1.9123	0.1578	0.0713
32	**5n**	−1.0120	1.5333	0.3472	1.9131	0.2218	0.0659
33	**5o**	−2.6170	1.3333	0.3407	1.9117	0.1409	0.0717
34	**5p**	−2.6980	1.3636	0.3408	1.9117	0.1409	0.0687
35	**5q**	−2.6320	1.3636	0.3346	1.9118	0.1471	0.0822
36	**5r**	−2.7160	1.4000	0.3412	1.9117	0.1406	0.0712
37	**6a**	−0.8850	1.6111	0.3569	1.9820	0.1729	0.1084
38	**6b**	−0.9660	1.6111	0.3574	1.9820	0.1703	0.1085
39	**6c**	−1.0120	1.6111	0.3547	1.9817	0.1736	0.1085
40	**6d**	−0.9120	1.6111	0.3571	1.9667	0.1726	0.1085
41	**6e**	−0.9870	1.6111	0.3558	1.9669	0.1705	0.1085
42	**6f**	−1.8640	1.5278	0.3577	1.9133	0.1700	0.1082
43	**6g**	−2.2020	1.4872	0.3573	1.9138	0.1690	0.0823
44	**6h**	−2.0990	1.4872	0.3579	1.9133	0.1698	0.0868
45	**6i**	−2.1350	1.4872	0.3590	1.9133	0.1699	0.0884
46	**6j**	−2.2100	1.5250	0.3665	1.9126	0.1701	0.0746
47	**6k**	−2.1330	1.5250	0.3569	1.9133	0.1742	0.0727
48	**6l**	−2.1880	1.5250	0.3632	1.9134	0.1692	0.0769
49	**6m**	−2.2390	1.4688	0.3344	1.9126	0.1702	0.0841
50	**6n**	−1.3850	1.5000	0.3468	1.9133	0.1689	0.1081
51	**6o**	−2.3840	1.3333	0.3340	1.9117	0.1719	0.0700
52	**6p**	−2.4250	1.3636	0.3340	1.9117	0.1720	0.0694

**Figure 3:**
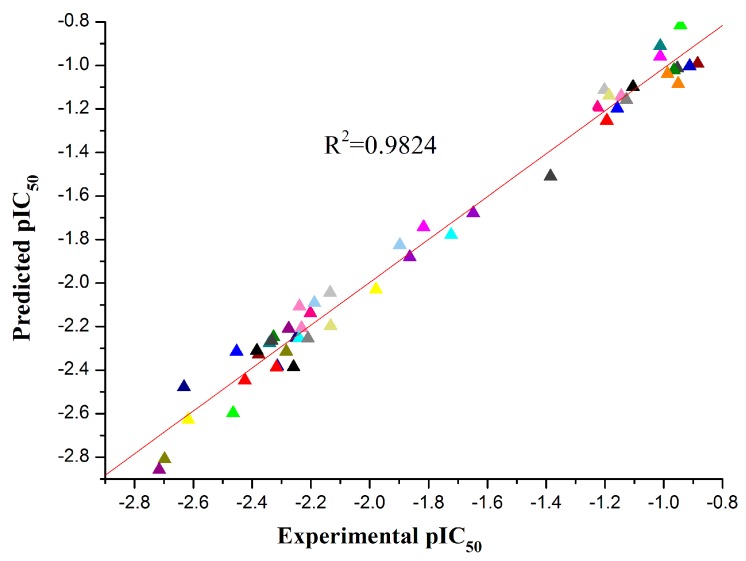
Experimental pIC_50_ *vs.* predicted pIC_50_.

**Figure 4:**
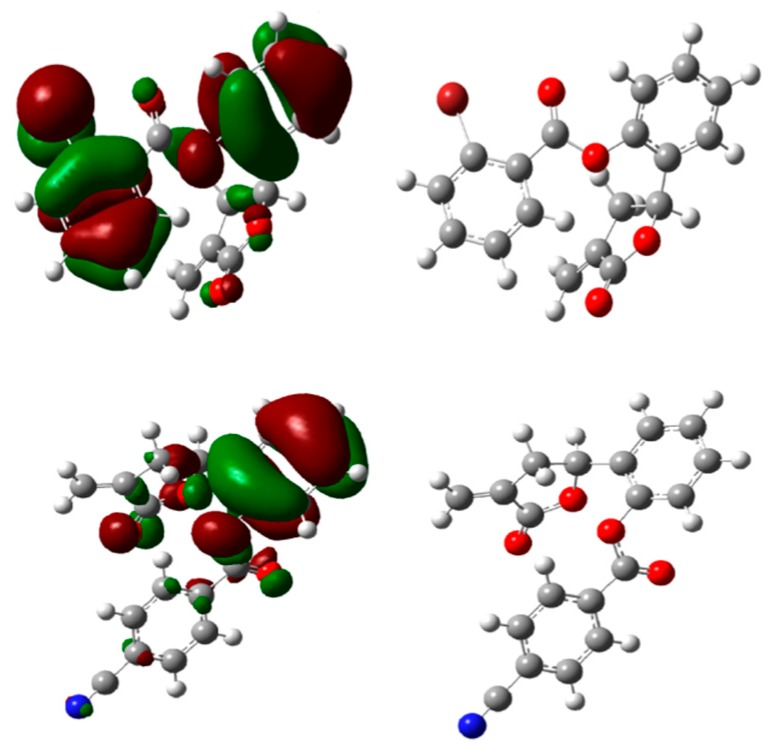
Optimized structures and HOMO energy maps for compounds **5c**,**f** from the DFT calculations of Gaussian 03W. The green parts represent positive molecular orbitals, and the red parts represent negative molecular orbitals.

The best statistical model for the pIC_50_ data had the following statistical characteristics: *R*^2^ = 0.9824, *F* = 203.01, *S*^2^ = 0.0083. This model included five descriptors in descending order according to their statistical significance (*t* values), which is shown in Table 3, and the regression coefficients *X* and their standard errors Δ*X* are also listed. The comparison between the experimental and predicted pIC_50_ is listed in Table 4, and the plot of the comparison between the predicted and experimental values is shown in Figure 3. The five descriptor QSAR model equation is described in the following Equation (2) (Figure 4):

pIC_50_ = −24.230 − 0.6261 × *N*_o_ − 10.225 × *q*^C^_max_ + 12.075 × MAOEP + 12.464 × *q*^H^_max_ + 16.086 × *q*^H^_min_ (2)

*N* = 52, *R*^2^ = 0.9824, *F* = 203.01, *S*^2^ = 0.0083.

**Table 3:** The best five-descriptor model.

Descriptor No.	*X*	±Δ*X*	*t*-Text	Descriptor
0	−2.4230 × 10	4.1535	−3.0433	Intercept
1	−6.2613 × 10^−1^	5.2603 × 10^−1^	−1.1903	*N*_o_^a^
2	−1.0225 × 10	2.9561	−3.4591	*q*^C^_max_^b^
3	1.2075 × 10	8.0165 × 10^−1^	15.0631	MAOEP ^c^
4	1.2464 × 10	2.0587	6.0544	*q*^H^_max_^d^
5	1.6086 × 10	1.8059	8.9076	*q*^H^_min_^e^

Note: ^a^ Number of occupied electronic levels of atoms; ^b^ Max. net atomic charge for a C atom; ^c^ Max. atomic orbital electronic population; ^d^ Max. net atomic charge for a H atom; ^e^ Min. net atomic charge for a C atom.

**Table 4:** The difference between the experimental pIC_50_ and predicted pIC_50_.

No.	Compd.	Calc. pIC_50_	Exp. pIC_50_	Difference	No.	Compd.	Calc. pIC_50_	Exp. pIC_50_	Difference
1	**4a**	−1.1403	−1.1450	0.0047	27	**5i**	−2.3856	−2.2590	−0.1266
2	**4b**	−1.0128	−0.9540	−0.0588	28	**5j**	−2.3869	−2.3170	−0.0699
3	**4c**	−1.0988	−1.1050	0.0062	29	**5k**	−2.5972	−2.4650	−0.1322
4	**4d**	−1.2533	−1.1940	−0.0593	30	**5l**	−2.3140	−2.4530	0.1390
5	**4e**	−0.8153	−0.9430	0.1277	31	**5m**	−2.2529	−2.2400	−0.0129
6	**4f**	−1.1974	−1.1580	−0.0394	32	**5n**	−0.9584	−1.0120	0.0536
7	**4g**	−1.7783	−1.7230	−0.0553	33	**5o**	−2.6263	−2.6170	−0.0093
8	**4h**	−1.7431	−1.8170	0.0739	34	**5p**	−2.8078	−2.6980	−0.1098
9	**4i**	−2.0285	−1.9790	−0.0495	35	**5q**	−2.4778	−2.6320	0.1542
10	**4j**	−2.3147	−2.2840	−0.0307	36	**5r**	−2.8579	−2.7160	−0.1419
11	**4k**	−2.2508	−2.2500	−0.0008	37	**6a**	−0.9917	−0.8850	−0.1067
12	**4l**	−2.2089	−2.2760	0.0671	38	**6b**	−1.0220	−0.9660	−0.0560
13	**4m**	−2.3273	−2.3780	0.0507	39	**6c**	−0.9105	−1.0120	0.1015
14	**4n**	−2.2477	−2.3270	0.0793	40	**6d**	−1.0028	−0.9120	−0.0908
15	**4o**	−2.2751	−2.3410	0.0659	41	**6e**	−1.0382	−0.9870	−0.0512
16	**4p**	−2.3821	−2.3150	−0.0671	42	**6f**	−1.8806	−1.8640	−0.0166
17	**4q**	−1.0839	−0.9510	−0.1329	43	**6g**	−2.1368	−2.2020	0.0652
18	**4r**	−1.6793	−1.6480	−0.0313	44	**6h**	−2.0781	−2.0990	0.0209
19	**5a**	−1.1940	−1.2250	0.0310	45	**6i**	−2.0438	−2.1350	0.0912
20	**5b**	−1.2255	−1.2490	0.0235	46	**6j**	−2.2545	−2.2100	−0.0445
21	**5c**	−1.1129	−1.2020	0.0891	47	**6k**	−2.1958	−2.1330	−0.0628
22	**5d**	−1.1579	−1.1270	−0.0309	48	**6l**	−2.0901	−2.1880	0.0979
23	**5e**	−1.1386	−1.1860	0.0474	49	**6m**	−2.1068	−2.2390	0.1322
24	**5f**	−1.8257	−1.8980	0.0723	50	**6n**	−1.5095	−1.3850	−0.1245
25	**5g**	−2.2060	−2.2320	0.0260	51	**6o**	−2.3109	−2.3840	0.0731
26	**5h**	−2.2643	−2.3340	0.0697	52	**6p**	−2.4467	−2.4250	−0.0217

The internal validation and the “leave-one-out” cross-validation methods were used to validate the developed QSAR model [28]. The internal validation was carried out by dividing the compound data into three subsets A–C, with **17**, **17** and **18** compounds respectively. The compounds **1**, **4**, **7**, **10**, *etc.*, went into the first subset (A); **2**, **5**, **8**, **11**, *etc.*, went into the second subset (B); and **3**, **6**, **9**, **12**, *etc.*, went into the third subset (C). Two of the three subsets, (A and B), (A and C), and (B and C), consist the training set while the remaining subset was treated as a test set. The correlation equations were derived from each of the training sets using the same descriptors and then used to predict values for the corresponding test set [29]. Internal validation results are presented in Table 5. The *R*_Training_^2^ and *R*_Test_^2^ are within 5% for all three sets, and the average values of *R*_Training_^2^ = 0.9833 and *R*_Test_^2^ = 0.9855 were close to the overall *R*^2^ value. Thus, the obtained QSAR model obtained demonstrated the predictive power of 3-fold cross-validation. Meanwhile, the “leave-one-out” method was completed in a similar manner to the internal validation. Every fourth compound (**1**, **5**, **9**, **13**, *etc.*) was put into an external test set, and the remaining compounds were left in the training set. The QSAR model containing the same five descriptors was obtained with *R*^2^ = 0.9862 from the training set. When the same QSAR model was applied on the test set, *R*^2^ = 0.9789 was observed. Therefore, the “leave-one out” cross-validation results were also satisfactory.

**Table 5:** Internal validation of the QSAR model ^a^.

Training Set	*N*	*R*^2^	*F*	*S*^2^	Test Set	*N*	*R*^2^	*F*	*S*^2^
A + B	34	0.9862	211.53	0.0095	C	18	0.9896	214.65	0.0086
B + C	35	0.9797	201.69	0.0112	A	17	0.9807	204.11	0.0104
A + C	35	0.9841	207.43	0.0090	B	17	0.9862	209.81	0.0092
Average		0.9833	206.83	0.0099	Average		0.9855	209.52	0.0094

Note: ^a^ Compounds A: **1**, **4**, **7**, **10**, **13**, **16**, **19**, **22**, **25**, **28**, **31**, **34**, **37**, **40**, **43**, **46**, **49**; Compounds B: **2**, **5**, **8**, **11**, **14**, **17**, **20**, **23**, **26**, **29**, **32**, **35**, **38**, **41**, **44**, **47**, **50**; Compounds C: **3**, **6**, **9**, **12**, **15**, **18**, **21**, **24**, **27**, **30**, **33**, **36**, **39**, **42**, **45**, **48**, **51**, **52**.

Descriptors involved in this model revealed the relationship between the compounds and the fungicidal activity. The 1st and 3rd most important descriptors obtained in the model were the number of occupied electronic levels of atoms and maximum atomic orbital electronic population, which belong to quantum-chemically descriptors and have a significant effect on the fungicidal activity. The number of occupied electronic levels of atoms depends directly on the quantum-chemically calculated charge distribution in the molecules, and therefore describes the polar interactions between molecules [30,31]. This study found that the derivatives with electron-withdrawing groups on the benzene ring showed higher *N*_o_ values than those with electron-donating groups. Maximum atomic orbital electronic population for a given atomic species in the molecule is an important index to describe the nucleophilicity of the molecule, which is directly related to molecular nucleophilic capacity and characterizes the susceptibility of the molecule to electrophilic attack [32]. In Equation (2), the maximum atomic orbital electronic population and pIC_50_ are positively correlated, which suggested that the electron withdrawing substitution groups of the derivatives are beneficial for the fungicidal activity against *C. lagenarium*. In fact, the α,β-unsaturated carbonyl system (Michael acceptor), which had higher electron deficiency induced by electron-withdrawing groups, can be easily attacked by bionucleophiles [33,34]. Therefore, the obtained QSAR study result partially met the above SAR study conclusion.

The 2nd, 4th and 5th descriptors obtained in the model were the maximum net atomic charge for a C atom, maximum net atomic charge for an H atom, and minimum net atomic charge for an H atom. These three descriptors belong to electrostatic descriptors, and they reflect characteristics of the charge distribution of the molecules [35,36]. Thus, the electrostatic descriptors play an important role in influencing the fungicidal activity of compounds. In Equation (2), appearance with a positive sign in the model indicated that a molecule with a higher descriptor value had a higher pIC_50_. On the contrary, a negative sign in the model indicated that a molecule with a lower descriptor value had a higher pIC_50_.

### 2.4. Cytotoxic Activity of the Representative Compounds against Human Tumor Cells Line (HepG2)

As a fact, compounds containing the α-methylene-γ-butyrolactone structure often exhibit a high toxicity potential against mammalian cells [37,38]. In order to ensure the selectivity of the fungicidal effects, the cytotoxicity of 24 representative derivatives was tested in a human tumor cells line (HepG2). The result is listed in Table 6, which indicated that the QSAR underlying the fungicidal and cytotoxic effects of these representative compounds are different. For instance, compound **6a** has the highest fungicidal activity with IC_50_ = 7.68 μM (against *C. lagenarium*) but moderate cytotoxic activity with IC_50_ = 30.2 μM (against HepG2 cell line), while, compound **4i** has low fungicidal activity with IC_50_ = 95.38 μM (against *C. lagenarium*) but high cytotoxic activity with IC_50_ = 5.3 μM (against HepG2 cell line). Through QSAR studies on fungicidal and antitumor activity of α-methylene-γ-butyrolactone derivatives, these are important points that need further investigation to seek high activity derivatives with weak cytotoxicity.

**Table 6:** *In vitro* fungicidal activity of compounds against *C. lagenarium* and cytotoxic activity against a human tumor cells line (HepG2).

No.	Compd.	IC_50_ (μM) (against *C. lagenarium*)	IC_50_ (μM) (against HepG2 Cell Line)
1	**4b**	8.99	22.4
2	**4e**	8.76	21.7
3	**4g**	52.87	18.3
4	**4i**	95.38	5.3
5	**4k**	177.96	28.5
6	**4o**	219.44	25.0
7	**4p**	206.51	19.5
8	**4q**	8.93	27.3
9	**5a**	16.77	28.4
10	**5d**	13.41	35.6
11	**5i**	181.69	85.2
12	**5l**	283.99	29.0
13	**5m**	173.74	23.8
14	**5p**	499.33	>131.7
15	**5r**	519.42	38.4
16	**6a**	7.68	30.2
17	**6d**	8.17	20.9
18	**6f**	73.14	18.5
19	**6g**	159.33	22.0
20	**6l**	154.27	58.6
21	**6n**	24.25	15.7
22	**6o**	242.03	>108.2
23	**4**	160.63	23.3
24	**6**	117.12	17.9

## 3. Materials and Methods

### 3.1. General Information

Chlorothalonil was purchased from Xiangtan Huayuan Fine-Chem Co. Ltd. (Xiangtan, China). 4-Dimethylaminopyridine (DMAP), *N*,*N*-dicyclohexylcarbodiimide (DCC) and carboxylic acids were purchased from J & K Chemical Ltd. (Beijing, China). Other reagents and solvents were obtained locally. All solvents were dried, and redistilled before use. The water used was redistilled and ion-free. Analytical thin-layer chromatography (TLC) was performed on silica gel GF_254_. Column chromatographic (CC) purification was carried out using silica gel (200–300 mesh). Above silica gel was obtained from Qingdao Haiyang Chemical Co., Ltd. (Qingdao, China). The melting points of the synthetic derivatives were determined on an *X*-6 apparatus (Beijing Tech., Beijing, China) and are uncorrected. Nuclear magnetic resonance (NMR) experiments were performed on an Avance 400/500 MHz instrument (Bruker, Bremerhaven, Germany). HR-MS (ESI) were obtained using a Bruker Apex-Ultra 7.0 T spectrometer. Reaction progress was monitored by thin-layer chromatography on silica gel GF-254 with detection by UV light.

### 3.2. Synthetic Procedures

#### 3.2.1. General Synthetic Procedure for the Intermediate Compounds

α-(Bromomethyl)acrylic acid was synthesized according to our previous report [21]. Hydroxybenzaldehyde (122.1 mg, 1.0 mmol), α-(bromomethyl) acrylic acid (198.0 mg, 1.2 mmol), and indium powder (136.0 mg, 1.2 mmol) were added to THF (10.0 mL) at room temperature. 6.0 M HCl was added to the above mixture when the starting aldehyde disappeared according to TLC analysis and stirring was continued for 6 hours. Then, the mixture was extracted with ethyl acetate (3 × 10 mL) and the organic phase dried over anhydrous Na_2_SO_4_ and evaporated under reduced pressure. The resulting residue was purified using preparative chromatography on silica gel eluting with 0%–40% ethyl acetate in petroleum ether. These intermediate compounds were used to prepare the target compounds.

*4-(4-Hydroxyphenyl)-2-methylenebutyrolactone* (**4**). White crystals; mp: 79–81 °C; 87% yield; ^1^H-NMR (400 MHz, CDCl_3_): δ 2.88 (ddt, 1H, *J* = 17.2, 6.5, 2.6 Hz, C*H*HC=CH_2_), 3.30 (ddt, 1H, *J =* 17.2, 8.0, 2.0 Hz, CH*H*C=CH_2_), 5.43 (t, 1H, *J =* 7.1 Hz, OCH), 5.70 (t, 1H, *J =* 2.1 Hz, C=C*H*H), 6.28 (t, 1H, *J =* 2.6 Hz, C=CH*H*), 6.84–7.11 (m, 4H, Ar*H*); ^13^C-NMR (100 MHz, CDCl_3_): δ 35.85, 79.14, 115.86, 123.05, 127.44, 130.59, 134.54, 156.76, 171.59; HR-MS (ESI): *m*/*z* calcd for C_11_H_11_O_3_ ([M + H]^+^) 191.0703, found 191.0703.

*4-(2-Hydroxyphenyl)-2-methylenebutyrolactone* (**5**)*.* Colourless oil; 81% yield; ^1^H-NMR (400 MHz, CDCl_3_): δ 2.95 (ddt, 1H, *J* = 17.4, 5.9, 2.7 Hz, C*H*HC=CH_2_), 3.42 (ddt, 1H, *J =* 17.4, 8.4, 2.4 Hz, CH*H*C=CH_2_), 5.66 (t, 1H, *J =* 2.4 Hz, C=C*H*H), 5.79 (dd, 1H, *J =* 8.3 6.1 Hz, OCH), 6.29 (t, 1H, *J =* 2.8 Hz, C=CH*H*), 6.86–7.23 (m, 4H, Ar*H*); ^13^C-NMR (100 MHz, CDCl_3_): δ 34.67, 76.23, 115.93, 120.13, 122.77, 126.04, 126.28, 129.65, 134.82, 153.67, 172.26; HR-MS (ESI): *m*/*z* calcd for C_11_H_11_O_3_ ([M + H]^+^) 191.0703, found 191.0703.

*4-(3-Hydroxyphenyl)-2-methylenebutyrolactone* (**6**)*.* White crystal ; mp: 78–79 °C; 81% yield; ^1^H-NMR (400 MHz, CDCl_3_): δ 2.82 (ddt, 1H, *J =* 17.2, 6.3, 2.8 Hz, C*H*HC=CH_2_), 3.30 (ddt, 1H, *J =* 17.2, 8.1, 2.3 Hz, CH*H*C=CH_2_), 5.41 (t, 1H, *J =* 7.4 Hz, OCH), 5.66 (t, 1H, *J =* 2.3 Hz, C=C*H*H), 6.25 (t, 1H, *J =* 2.7 Hz, C=CH*H*), 6.76–7.19 (m, 4H, Ar*H*); ^13^C-NMR (100 MHz, CDCl_3_): δ 35.91, 78.62, 112.44, 115.87, 117.09, 123.31, 130.16, 134.04, 141.10, 156.71, 171.55; HR-MS (ESI): *m*/*z* calcd for C_11_H_11_O_3_ ([M + H]^+^) 191.0703, found 191.0703.

#### 3.2.2. General Synthetic Procedure for Ester Compounds

4-Dimethylaminopyridine (DMAP, 30.0 mg, 0.2 mmol) and the appropriate intermediate compounds 4, 5 or 6 (196.0 mg, 1.1 mmol) were added to anhydrous CH_2_Cl_2_ (15.0 mL) containing the respective carboxylic acid (1.1 mmol). Then the mixture was cooled to 0 °C. *N*,*N*-dicyclohexyl-carbodiimide (DCC, 226.0 mg, 1.1 mmol) dissolved in anhydrous CH_2_Cl_2_ (10.0 mL) was added dropwise into the mixture over a period of 10 min at 0 °C and the mixture was then stirred at room temperature until the reaction was complete according to the TLC analysis. Then, the mixture was filtered. Finally, the residual organic layers were extracted by ethyl acetate (3 × 30 mL) and dried over anhydrous Na_2_SO_4_. After filtering, the solution was evaporated under vacuum. The target compounds were purified by column chromatography on silica gel eluting with 0%–40% ethyl acetate in petroleum ether. The structures of all ester derivatives were characterized by ^1^H-NMR, ^13^C-NMR, and HR-ESI-MS, and the data are listed below.

*4-[4-(2-Chlorobenzoyloxy)phenyl]-2-methylenebutyrolactone* (**4a**) White solid; mp: 187.2–187.9 °C; 40% yield; ^1^H-NMR (400 MHz, CDCl_3_): δ 8.05 (dd, *J* =7.8, 1.1 Hz, 1H, Ar*H*), 7.56–7.47 (m, 3H, Ar*H*), 7.44–7.37 (m, 4H, Ar*H*), 6.33 (t, *J* = 2.8 Hz, 1H, C=C*H*H), 5.61–5.52 (m, 1H, OC*H*), 5.56 (d, *J* = 7.7 Hz, 1H, C=CH*H*), 3.50–3.37 (m, 1H, C*H*HC=CH_2_), 3.04–2.82 (m, 1H, CH*H*C=CH_2_); ^13^C-NMR (125 MHz, CDCl_3_): δ 170.06, 163.97, 150.70, 137.72, 134.50, 133.94, 133.41, 131.97, 131.44, 128.99, 126.77, 122.83, 122.16, 77.62, 77.05, 76.73, 36.36, 1.05; HR-MS (ESI): *m*/*z* calcd for C_18_H_13_ClNaO_4_ ([M + Na]^+^) 351.0394, found 351.0395.

*4-[4-(3-Chlorobenzoyloxy)phenyl]-2-methylenebutyrolactone* (**4b**) White solid; mp: 168.8–169.2 °C; 54% yield; ^1^H-NMR (500 MHz, CDCl_3_): δ 8.24 (m, 1H, Ar*H*), 7.69–7.56 (m, 1H, Ar*H*), 7.54–7.34 (m, 3H, Ar*H*), 7.31–7.19 (m, 3H, Ar*H*), 6.37 (dd, *J* = 14.9, 12.1 Hz, 1H, C=C*H*H), 5.76 (dd, *J* = 14.6, 12.2 Hz, 1H, OC*H*), 5.60 (dd, *J* = 20.0, 12.6 Hz, 1H, C=CH*H*), 3.54–3.37 (m, 1H, C*H*HC=CH_2_), 3.02–2.88 (m, 1H, CH*H*C=CH_2_); ^13^C-NMR (125 MHz, CDCl_3_): δ 150.76, 137.71, 134.84, 133.86, 131.05, 130.22, 129.95, 128.31, 126.75, 122.74, 122.02, 77.31, 77.01, 76.75, 36.30, 29.70; HR-MS (ESI): *m*/*z* calcd for C_18_H_13_ClNaO_4_ ([M + Na]^+^) 351.0394, found 351.0395.

*4-[4-(4-Chlorobenzoyloxy)phenyl]-2-methylenebutyrolactone* (**4c**) White solid; mp: 171.3–171.6 °C; 65% yield; ^1^H-NMR (400 MHz, CDCl_3_): δ 8.30–8.02 (m, 2H, Ar*H*), 7.68–7.14 (m, 6H, Ar*H*), 6.33 (t, *J* = 2.8 Hz, 1H, C=C*H*H), 5.72 (t, *J* = 2.5 Hz, 1H, OC*H*), 5.60–5.47 (m, 1H, C=CH*H*), 3.43 (ddt, *J* = 17.1, 8.0, 2.4 Hz, 1H, C*H*HC=CH_2_), 2.94 (ddt, *J* = 17.1, 6.1, 2.9 Hz, 1H, CH*H*C=CH_2_); ^13^C-NMR (125 MHz, CDCl_3_): δ 170.01, 150.82, 140.37, 137.63, 133.95, 131.59, 129.04, 126.73, 122.80, 122.16, 77.39, 77.04, 76.72, 36.31. HR-MS (ESI): *m*/*z* calcd for C_18_H_13_ClNaO_4_ ([M + Na]^+^) 351.0394, found 351.0395.

*4-[4-(2-Bromobenzoyloxy)phenyl]-2-methylenebutyrolactone* (**4d**) White crystal; mp: 174.5–174.9 °C; 62% yield; ^1^H-NMR (500 MHz, CDCl_3_): δ 8.04 (dd, *J* = 7.5, 1.7 Hz, 1H, Ar*H*), 7.91–7.68 (m, 1H, Ar*H*), 7.56–7.08 (m, 6H, Ar*H*), 6.36 (t, *J* = 2.8 Hz, 1H, C=C*H*H), 5.74 (t, *J* = 2.4 Hz, 1H, OC*H*), 5.61–5.50 (m, 1H, C=CH*H*), 3.56–3.08 (m, 1H, C*H*HC=CH_2_), 3.05–2.67 (m, 1H, CH*H*C=CH_2_). ^13^C-NMR (125 MHz, CDCl_3_): δ 134.69, 133.30, 131.85, 127.36, 126.70, 122.73, 122.03, 77.32, 77.01, 76.75, 36.35, 29.70, 14.99.HR-MS (ESI): *m*/*z* calcd for C_18_H_13_BrNaO_4_ ([M + Na]^+^) 394.9890, found 394.9889.

*4-[4-(3-Bromobenzoyloxy)phenyl]-2-methylenebutyrolactone* (**4e**) White crystal; mp: 176.5–176.8 °C; 45% yield; ^1^H-NMR (500 MHz, CDCl_3_): δ 8.37 (s, 1H, Ar*H*), 8.16 (d, *J* = 7.7 Hz, 1H, Ar*H*), 7.80 (d, *J* = 7.9 Hz, 1H, Ar*H*), 7.56–7.19 (m, 4H, Ar*H*), 6.36 (t, *J* = 2.8 Hz, 1H, C=C*H*H), 5.75 (t, *J* = 2.4 Hz, 1H, OC*H*), 5.66–5.52 (m, 1H, C=CH*H*), 5.42 (dd, *J* = 10.6, 5.3 Hz, 1H, Ar*H*), 3.57–3.37 (m, 1H, C*H*HC=CH_2_), 2.95 (s, 1H, CH*H*C=CH_2_); ^13^C-NMR (125 MHz, CDCl_3_): δ 136.73, 133.16, 132.05, 131.69, 130.22, 128.79, 126.91, 126.53, 122.77, 122.20, 78.63, 77.34, 77.03, 76.78, 40.03, 36.36, 29.72, 15.00. HR-MS (ESI): *m*/*z* calcd for C_18_H_13_BrNaO_4_ ([M + Na]^+^) 394.9890, found 394.9889.

*4-[4-(4-Bromobenzoyloxy)phenyl]-2-methylenebutyrolactone* (**4f**) White crystal; mp: 175.1–175.6 °C; 52% yield; ^1^H-NMR (500 MHz, CDCl_3_): δ 8.10 (d, *J* = 8.5 Hz, 2H, Ar*H*), 7.72 (t, *J* = 12.5 Hz, 4H, Ar*H*), 6.38 (t, *J* = 2.8 Hz, 1H, C=C*H*H), 5.76 (t, *J* = 2.4 Hz, 1H, OC*H*), 5.65–5.54 (m, 1H, C=CH*H*), 5.43 (dd, *J* = 10.7, 5.2 Hz, 2H, Ar*H*), 3.55–3.36 (m, 1H, C*H*HC=CH_2_), 3.04–2.93 (m, 1H, CH*H*C=CH_2_); ^13^C-NMR (125 MHz, CDCl_3_): δ 150.78, 136.95, 132.05, 131.69, 129.05, 128.28, 126.76, 122.75, 122.08, 78.62, 77.28, 77.03, 76.78, 40.03, 36.36, 15.00. HR-MS (ESI): *m*/*z* calcd for C_18_H_13_BrNaO_4_ ([M + Na]^+^) 394.9890, found 394.9889.

*4-[4-(3-Benzonitrile)phenyl]-2-methylenebutyrolactone* (**4g**) Yellow oil; 54% yield; ^1^H-NMR (500 MHz, CDCl_3_): δ 8.52 (s, 1H, Ar*H*), 8.45 (d, *J* = 8.0 Hz, 1H, Ar*H*), 7.95 (d, *J* = 7.8 Hz, 1H, Ar*H*), 7.70 (t, *J* = 7.9 Hz, 1H, Ar*H*), 7.29 (t, *J* = 4.3 Hz, 4H, Ar*H*), 6.36 (t, *J* = 2.8 Hz, 1H, 1H, C=C*H*H), 5.75 (t, *J* = 2.4 Hz, 1H, OC*H*), 5.65–5.56 (m, 1H, C=CH*H*), 3.46 (ddd, *J* = 10.5, 5.7, 2.4 Hz, 1H, C*H*HC=CH_2_), 2.96 (ddt, *J* = 17.0, 6.1, 2.9 Hz, 1H, CH*H*C=CH_2_), ^13^C-NMR (125 MHz, CDCl_3_): δ 163.13, 150.51, 138.01, 136.69, 134.16, 133.82, 130.69, 129.73, 126.83, 122.82, 121.90, 113.36, 77.26, 77.01, 76.76, 49.17, 36.29, 33.95, 25.62. HR-MS (ESI): *m*/*z* calcd for C_19_H_13_NNaO_4_ ([M + Na]^+^) 342.0736, found 342.0740.

*4-[4-(4-Benzonitrile)phenyl]-2-methylenebutyrolactone* (**4h**) Yellow oil; 57% yield; ^1^H-NMR (500 MHz, CDCl_3_): δ 8.51–8.39 (m, 1H, Ar*H*), 8.34 (t, *J* = 7.3 Hz, 2H, Ar*H*), 7.98 (d, *J* = 7.8 Hz, 1H, Ar*H*), 7.85 (s, 2H, Ar*H*), 7.73 (t, *J* = 7.9 Hz, 1H, Ar*H*), 6.37 (t, *J* = 2.8 Hz, 1H, C=C*H*H), 6.25 (t, *J* = 2.9 Hz, 1H, Ar*H*), 5.75 (t, *J* = 2.4 Hz, 1H, OC*H*), 5.71–5.64 (m, 2H, Ar*H*), 5.61–5.55 (m, 1H, C=CH*H*), 3.49–3.41 (m, 1H, C*H*HC=CH_2_), 3.38–3.28 (m, 1H, CH*H*C=CH_2_). ^13^C-NMR (125 MHz, CDCl_3_): δ 137.00, 133.71, 132.43, 130.67, 129.88, 127.05, 126.80, 123.06, 122.84, 121.89, 77.26, 77.01, 76.75, 73.73, 49.17, 36.28, 33.95, 29.69, 25.64. HR-MS (ESI): *m*/*z* calcd for C_19_H_13_NNaO_4_ ([M + Na]^+^) 342.0736, found 342.0740.

*4-(4-Benzoyloxyphenyl)-2-methylenebutyrolactone* 4-(4-Benzoyloxyphenyl)-2-methylenebutyrolactone (**4i**) White solid; mp: 223.3–223.7 °C; 55% yield; ^1^H-NMR (500 MHz, CDCl_3_): δ 8.25 (d, *J* = 8.3 Hz, 5H, Ar*H*), 7.70 (t, *J* = 7.4 Hz, 4H, Ar*H*), 6.38 (s, 1H, C=C*H*H), 5.76 (d, *J* = 2.4 Hz, 1H, OC*H*), 5.63–5.55 (m, 1H, C=CH*H*), 4.11 (dd, *J* = 15.3, 7.9 Hz, 1H, C*H*HC=CH_2_), 3.47 (dd, *J* = 17.1, 8.1 Hz, 1H, CH*H*C=CH_2_); ^13^C-NMR (125 MHz, CDCl_3_): δ 133.76, 130.21, 128.64, 126.72, 122.12, 78.70, 77.28, 77.03, 76.77, 40.04, 36.41, 29.72, 15.00. HR-MS (ESI): *m*/*z* calcd for C_18_H_14_NaO_4_ ([M + Na]^+^) 317.0784, found 317.0788.

*4-[4-(2-Methylbenzoyloxy)phenyl]-2-methylenebutyrolactone* (**4j**) White crystals; mp: 189.6–190.1 °C; 53% yield; ^1^H-NMR (500 MHz, CDCl_3_): δ 8.18 (d, *J* = 7.8 Hz, 1H, Ar*H*), 7.58–7.46 (m, 1H, Ar*H*), 7.40 (t, *J* = 14.7 Hz, 2H, Ar*H*), 7.35 (t, *J* = 7.7 Hz, 2H, Ar*H*), 7.28 (t, *J* = 6.3 Hz, 2H, Ar*H*), 6.35 (t, *J* = 2.8 Hz, 1H, C=C*H*H), 5.73 (t, *J* = 2.4 Hz, 1H, C=CH*H*), 5.64–5.40 (m, 1H, OC*H*), 3.45 (ddt, *J* = 17.1, 8.0, 2.4 Hz, 1H, C*H*HC=CH_2_), 3.05–2.85 (m, 1H, CH*H*C=CH_2_), 2.70 (s, 3H, ArC*H*_3_); ^13^C-NMR (125 MHz, CDCl_3_): δ 170.00, 165.67, 151.00, 141.44, 137.39, 134.02, 132.91, 126.65, 125.97, 125.11, 122.96, 122.66, 122.35, 77.47, 77.30, 77.05, 76.79, 36.27, 21.95; HR-MS (ESI): *m*/*z* calcd for C_19_H_16_NaO_4_ ([M + Na]^+^) 331.0940, found 331.0937.

*4-[4-(3-Methylbenzoyloxy)phenyl]-2-methylenebutyrolactone* (**4k**) White crystals; mp: 186.2–186.6 °C; 52% yield; ^1^H-NMR (500 MHz, CDCl_3_): δ 8.00 (d, *J* = 8.9 Hz, 2H, Ar*H*), 7.57–7.14 (m, 6H, Ar*H*), 6.33 (t, *J* = 2.8 Hz, 1H, C=C*H*H), 5.72 (t, *J* = 2.5 Hz, 1H, C=CH*H*), 5.58–5.50 (m, 1H, OC*H*), 3.43 (ddt, *J* = 17.1, 8.0, 2.4 Hz, 1H, C*H*HC=CH_2_), 3.05–2.82 (m, 1H, CH*H*C=CH_2_), 2.45 (s, 3H, ArCH_3_); ^13^C-NMR (125 MHz, CDCl_3_): δ 170.03, 165.28, 151.07, 138.51, 137.37, 134.55, 134.01, 130.71, 129.19, 128.53, 127.37, 126.67, 122.26, 77.48, 77.28, 77.02, 76.77, 36.32, 21.30. HR-MS (ESI): *m*/*z* calcd for C_19_H_16_NaO_4_ ([M + Na]^+^) 331.0940, found 331.0937.

*4-[4-(4-Methylbenzoyloxy)phenyl]-2-methylenebutyrolactone* (**4l**) White crystals; mp: 184.5–185.1 °C; 60% yield; ^1^H-NMR (500 MHz, CDCl_3_): δ 8.11 (d, *J* = 8.2 Hz, 2H, Ar*H*), 7.56–7.19 (m, 6H, Ar*H*), 6.35 (t, *J* = 2.8 Hz, 1H, C=C*H*H), 5.74 (t, *J* = 2.5 Hz, 1H, C=CH*H*), 5.64–5.40 (m, 1H, OC*H*), 3.45 (ddt, *J* = 17.1, 8.0, 2.4 Hz, 1H, C*H*HC=CH_2_), 3.08–2.82 (m, 1H, CH*H*C=CH_2_), 2.48 (s, 3H, ArCH_3_); ^13^C-NMR (125 MHz, CDCl_3_): δ 169.99, 165.13, 151.11, 144.63, 138.51, 137.30, 134.03, 130.24, 129.34, 127.37, 126.58, 122.65, 122.27, 77.48, 77.27, 77.02, 76.76, 36.32, 21.77. HR-MS (ESI): *m*/*z* calcd for C_19_H_16_NaO_4_ ([M + Na]^+^) 331.0940, found 331.0937.

*4-[4-(2-Methoxylbenzoyloxy)phenyl]-2-methylenebutyrolactone* (**4m**) White crystals; mp: 179.8–180.4 °C; 60% yield; ^1^H-NMR (500 MHz, CDCl_3_): δ 8.02 (dd, *J* = 8.0, 1.7 Hz, 1H, Ar*H*), 7.56 (td, *J* = 8.2, 1.8 Hz, 1H, Ar*H*), 7.40–6.97 (m, 6H, Ar*H*), 6.32 (t, *J* = 2.8 Hz, 1H, C=C*H*H), 5.71 (t, *J* = 2.5 Hz, 1H, C=CH*H*), 5.59–5.48 (m, 1H, OC*H*), 3.94 (s, 3H, ArOC*H*_3_), 3.42 (ddt, *J* = 17.1, 8.0, 2.4 Hz, 1H, C*H*HC=CH_2_), 2.93 (ddt, *J* = 9.3, 6.0, 2.9 Hz, 1H, CH*H*C=CH_2_); ^13^C-NMR (125 MHz, CDCl_3_): δ 170.08, 164.25, 159.97, 151.08, 137.20, 134.54, 134.06, 132.25, 126.56, 122.67, 122.37, 120.25, 112.25, 77.55, 77.30, 77.04, 76.79, 56.07, 36.34; HR-MS (ESI): *m*/*z* calcd for C_19_H_16_NaO_5_ ([M + Na]^+^) 347.0889, found 347.0891.

*4-[4-(3-Methoxylbenzoyloxy)phenyl]-2-methylenebutyrolactone* (**4n**) White crystals; mp: 185.7–186.2 °C; 44% yield; ^1^H-NMR (500 MHz, CDCl_3_): δ 7.85 (d, *J* = 7.7 Hz, 1H, Ar*H*), 7.74 (s, 4H, Ar*H*), 6.38 (s, 1H, C=C*H*H), 5.76 (s, 1H, C=CH*H*), 5.66–5.56 (m, 1H, OC*H*), 5.44 (dd, *J* = 10.7, 5.2 Hz, 3H, Ar*H*), 4.35 (s, 3H, ArOC*H*_3_), 4.11 (dd, *J* = 17.0, 9.5 Hz, 1H, C*H*HC=CH_2_), 3.47 (dd, *J* = 17.1, 8.1 Hz, 1H, CH*H*C=CH_2_); ^13^C-NMR (125 MHz, CDCl_3_): δ 170.03, 164.28, 159.77, 129.67, 126.76, 122.64, 122.10, 120.32, 114.60, 78.69, 77.28, 77.03, 76.78, 55.56, 40.04, 36.41, 29.72, 15.00. HR-MS (ESI): *m*/*z* calcd for C_19_H_16_NaO_5_ ([M + Na]^+^) 347.0889, found 347.0891.

*4-[4-(4-Methoxylbenzoyloxy)phenyl]-2-methylenebutyrolactone* (**4o**) White crystals; mp: 184.5–185.2 °C; 51% yield; ^1^H-NMR (500 MHz, CDCl_3_): δ 8.28–8.00 (m, 2H, Ar*H*), 7.53–7.30 (m, 2H, Ar*H*), 7.28–7.14 (m, 2H, Ar*H*), 7.02–6.95 (m, 2H, Ar*H*), 6.32 (t, *J* = 2.8 Hz, 1H, C=C*H*H), 5.71 (t, *J* = 2.5 Hz, 1H, C=CH*H*), 5.58–5.41 (m, 1H, OC*H*), 3.90 (s, 3H, ArOC*H*_3_), 3.49–3.33 (m, 1H, C*H*HC=CH_2_), 2.94 (ddd, *J* = 14.2, 6.3, 3.1 Hz, 1H, CH*H*C=CH_2_);^13^C-NMR (125 MHz, CDCl_3_): δ 170.08, 164.83, 164.05, 159.97, 151.16, 137.23, 134.05, 132.36, 126.64, 122.70, 122.33, 121.55, 113.92, 77.46, 77.06, 76.74, 55.56, 36.32. HR-MS (ESI): *m*/*z* calcd for C_19_H_16_NaO_5_ ([M + Na]^+^) 347.0889, found 347.0891.

*4-[4-(Propionyloxy)phenyl]-2-methylenebutyrolactone* (**4p**) Colourless oil; 68% yield; ^1^H-NMR (500 MHz, CDCl_3_): δ 6.35 (t, *J* = 2.8 Hz, 1H, C=C*H*H), 5.74 (t, *J* = 2.4 Hz, 1H, C=CH*H*), 5.63–5.46 (m, 1H, OC*H*), 5.39 (dd, *J* = 10.7, 5.2 Hz, 3H,CH_3_CH_2_), 3.44 (dd, *J* = 17.1, 8.1 Hz, 1H, C*H*HC=CH_2_), 3.03–2.90 (m, 1H, CH*H*C=CH_2_), 1.67 (s, 2H,CH_3_CH_2_); ^13^C-NMR (125 MHz, CDCl_3_): δ 178.99, 172.87, 150.84, 136.53, 126.63, 122.64, 78.68, 77.39, 77.06, 76.81, 39.98, 36.33, 27.75, 14.97. HR-MS (ESI): *m*/*z* calcd for C_14_H_14_NaO_4_ ([M + Na]^+^) 269.0784, found 269.0786.

*[4-(4-Hydroxy-3-methoxycinnamoyloxy)phenyl]-2-methylenebutyrolactone* (**4q**) Yellow oil; 45% yield; ^1^H-NMR (400 MHz, CDCl_3_): δ 7.80 (d, *J* = 15.9 Hz, 1H, ArOH), 7.36 (d, *J* = 8.6 Hz, 2H, Ar*H*), 7.21 (dd, *J* = 21.2, 12.6 Hz, 2H, Ar*H*), 7.16–7.05 (m, 2H, CH=CH), 6.95 (d, *J* = 8.2 Hz, 1H, Ar*H*), 6.47 (d, *J* = 15.9 Hz, 1H, Ar*H*), 6.32 (t, *J* = 2.8 Hz, 1H, C=C*H*H), 6.10 (s, 1H, Ar*H*), 5.71 (t, *J* = 2.5 Hz, 1H, C=CH*H*), 5.58–5.44 (m, 1H, OC*H*),4.02–3.82 (m, 3H, ArOC*H*_3_), 3.49–3.29 (m, 1H, C*H*HC=CH_2_), 2.93 (ddt, *J* = 12.2, 6.0, 2.9 Hz, 1H, CH*H*C=CH_2_); ^13^C-NMR (125 MHz, CDCl_3_): δ 170.02, 165.56, 151.01, 148.60, 147.01, 137.172, 134.06, 126.63, 123.51, 122.18, 114.92, 114.22, 109.66, 76.72, 56.01, 49.16, 36.28, 33.94, 30.90, 29.69, 25.62. HR-MS (ESI): *m*/*z* calcd for C_21_H_18_NaO_6_ ([M + Na]^+^) 389.0997, found 389.0995.

*4-[4-(Cinnamoyloxy)phenyl]-2-methylenebutyrolactone* (**4r**) Yellow oil; 43% yield; ^1^H-NMR (400 MHz, CDCl_3_): δ 7.88 (d, *J* = 16.0 Hz, 1H, Ar*H*), 7.64–7.53 (m, 2H, Ar*H*), 7.47–7.40 (m, 2H, Ar*H*), 7.39–7.32 (m, 2H, Ar*H*), 7.28–7.16 (m, 2H, CH=CH), 6.63 (d, *J* = 16.0 Hz, 1H, Ar*H*), 6.32 (t, *J* = 2.8 Hz, 1H, C=C*H*H), 5.71 (t, *J* = 2.5 Hz, 1H, C=CH*H*), 5.63–5.46 (m, 1H, OC*H*), 3.41 (ddt, *J* = 17.1, 8.0, 2.4 Hz, 1H, C*H*HC=CH_2_), 2.93 (ddt, *J* = 9.4, 6.0, 2.9 Hz, 1H, CH*H*C=CH_2_); ^13^C-NMR (100 MHz, CDCl_3_): δ 170.06, 165.31, 150.88, 146.99, 137.31, 134.05, 130.86, 129.05, 128.37, 126.65, 122.72, 122.16, 116.99, 77.44, 77.06, 76.74, 36.30, 33.94, 30.91, 29.63. HR-MS (ESI): *m*/*z* calcd for C_20_H_16_NaO_4_ ([M + Na]^+^) 343.0940, found 343.0941.

*4-[2-(3-Chlorobenzoyloxy)phenyl]-2-methylenebutyrolactone* (**5a**) White solid; mp: 201.3–201.8 °C; 54% yield; ^1^H-NMR (500 MHz, CDCl_3_): δ 8.16 (t, *J* = 1.8 Hz, 1H, Ar*H*), 8.08 (d, *J* = 7.8 Hz, 1H, Ar*H*), 7.71–7.59 (m, 1H, Ar*H*), 7.53–7.45 (m, 2H, Ar*H*), 7.43 (dt, *J* = 7.8, 3.9 Hz, 1H, Ar*H*), 7.34 (dd, *J* = 9.4, 4.9 Hz, 1H, Ar*H*), 7.21 (dd, *J* = 8.0, 0.7 Hz, 1H, Ar*H*), 6.24 (t, *J* = 2.9 Hz, 1H, C=C*H*H), 5.68 (dd, *J* = 8.4, 6.2 Hz, 1H, C=CH*H*), 5.63 (t, *J* = 2.5 Hz, 1H, OC*H*), 3.32 (ddt, *J* = 17.4, 8.4, 2.6 Hz, 1H, C*H*HC=CH_2_), 2.90 (ddt, *J* = 17.4, 5.9, 2.9 Hz, 1H, CH*H*C=CH_2_); ^13^C-NMR (125 MHz, CDCl_3_): δ 169.96, 163.75, 147.46, 135.04, 134.19, 133.51, 132.32, 130.46, 129.67, 128.34, 126.85, 126.40, 123.09, 122.85, 77.31, 77.06, 76.80, 35.20; HR-MS (ESI): *m*/*z* calcd for C_18_H_14_ClO_4_ ([M + Na]^+^) 325.0574, found 325.0575.

*4-[2-(4-Chlorobenzoyloxy)phenyl]-2-methylenebutyrolactone* (**5b**) White solid; mp: 208.7–209.3 °C; 48% yield; ^1^H-NMR (400 MHz, CDCl_3_): δ 8.17–8.07 (m, 2H, Ar*H*), 7.59–7.45 (m, 4H, Ar*H*), 7.26 (s, 2H, Ar*H*), 6.22 (s, 1H, C=C*H*H), 5.67 (dd, *J* = 8.4, 6.1 Hz, 1H, OC*H*), 5.61 (s, 1H, C=CH*H*), 3.30 (ddt, *J* = 17.4, 8.4, 2.6 Hz, 1H, C*H*HC=CH_2_), 2.90 (ddt, *J* = 17.4, 5.9, 2.9 Hz, 1H, CH*H*C=CH_2_); ^13^C-NMR (125 MHz, CDCl_3_): δ 168.96, 165.75, 146.45, 137.04, 134.29, 131.58, 129.66, 129.25, 128.81, 128.26, 126.76, 126.40, 123.09, 122.97, 77.35, 77.03, 76.72, 30.87. HR-MS (ESI): *m*/*z* calcd for C_18_H_14_ClO_4_ ([M + Na]^+^) 325.0572, found 325.0575.

*4-[2-(2-Bromobenzoyloxy)phenyl]-2-methylenebutyrolactone* (**5c**) White crystal; mp: 204.6–205.1 °C; 43% yield; ^1^H-NMR (500 MHz, CDCl_3_): δ 8.05 (dd, *J* = 7.5, 1.9 Hz, 1H, Ar*H*), 7.79 (dd, *J* = 7.7, 1.2 Hz, 1H, Ar*H*), 7.56–7.16 (m, 6H, Ar*H*), 6.25 (t, *J* = 2.8 Hz, 1H, C=C*H*H), 5.76 (dd, *J* = 8.3, 6.2 Hz, 1H, OC*H*), 5.66 (t, *J* = 2.5 Hz, 1H, C=CH*H*), 3.60–3.22 (m, 1H, C*H*HC=CH_2_), 2.93 (ddt, *J* = 17.4, 5.9, 2.8 Hz, 1H, CH*H*C=CH_2_); ^13^C-NMR (125 MHz, CDCl_3_): δ 164.19, 147.43, 134.88, 132.39, 131.97, 130.59, 129.59, 127.57, 126.81, 126.29, 122.88, 122.69, 122.42, 77.28, 77.03, 76.78, 73.60, 35.33.HR-MS (ESI): *m*/*z* calcd for C_18_H_14_BrO_4_ ([M + H]^+^) 373.0070, found 373.0072.

*4-[2-(3-Bromobenzoyloxy)phenyl]-2-methylenebutyrolactone* (**5d**) White crystals; mp: 189.7–190.4 °C; 45% yield; ^1^H-NMR (500 MHz, CDCl_3_): δ 8.34 (t, *J* = 1.6 Hz, 1H, Ar*H*), 8.14 (d, *J* = 7.8 Hz, 1H, Ar*H*), 7.91–7.76 (m, 1H, Ar*H*), 7.54–7.40 (m, 3H, Ar*H*), 7.36 (td, *J* = 7.6, 0.7 Hz, 1H, Ar*H*), 7.23 (dd, *J* = 8.0, 0.6 Hz, 1H, Ar*H*), 6.25 (t, *J* = 2.9 Hz, 1H, C=C*H*H), 5.69 (dd, *J* = 8.3, 6.2 Hz, 1H, OC*H*), 5.65 (t, *J* = 2.5 Hz, 1H, C=CH*H*), 3.33 (ddt, *J* = 17.4, 8.4, 2.5 Hz, 1H, C*H*HC=CH_2_), 2.92 (ddt, *J* = 17.4, 5.9, 2.8 Hz, 1H, CH*H*C=CH_2_); ^13^C-NMR (125 MHz, CDCl_3_): δ 169.89, 163.60, 147.50, 137.07, 133.54, 133.12, 130.69, 130.42, 129.65, 128.77, 126.83, 126.42, 123.15, 77.32, 77.06, 76.81, 73.61, 35.20. HR-MS (ESI): *m*/*z* calcd for C_18_H_14_BrO_4_ ([M + H]^+^) 373.0070, found 373.0069.

*4-[2-(4-Bromobenzoyloxy)phenyl]-2-methylenebutyrolactone* (**5e**) White crystals; mp: 184.7–185.2 °C; 53% yield; ^1^H-NMR (500 MHz, CDCl_3_): δ 8.05 (dd, *J* = 23.8, 8.5 Hz, 2H, Ar*H*), 7.70 (t, *J* = 11.9 Hz, 2H, Ar*H*), 7.56–7.20 (m, 4H, Ar*H*), 6.25 (t, *J* = 2.8 Hz, 1H, C=C*H*H), 5.69 (dd, *J* = 8.3, 6.2 Hz, 1H, OC*H*), 5.63 (t, *J* = 2.4 Hz, 1H, C=CH*H*), 3.32 (ddt, *J* = 17.4, 8.4, 2.5 Hz, 1H, C*H*HC=CH_2_), 2.92 (ddt, *J* = 17.4, 5.9, 2.8 Hz, 1H, CH*H*C=CH_2_); ^13^C-NMR (125 MHz, CDCl_3_): δ 169.90, 164.22, 147.58, 133.55, 132.27, 131.65, 130.89, 130.62, 129.58, 127.66, 126.75, 126.44, 122.93, 77.27, 77.02, 76.76, 73.69, 35.16. HR-MS (ESI): *m*/*z* calcd for C_18_H_14_BrO_4_ ([M + H]^+^) 373.0070, found 373.0069.

*4-[2-(4-Benzonitrile)phenyl]-2-methylenebutyrolactone* (**5f**) Yellow oil; 65% yield; ^1^H-NMR (500 MHz, CDCl_3_): δ 8.31 (d, *J* = 8.5 Hz, 2H, Ar*H*), 7.86 (d, *J* = 8.5 Hz, 2H, Ar*H*), 7.55–7.43 (m, 2H, Ar*H*), 7.41–7.35 (m, 1H, Ar*H*), 7.30–7.24 (m, 1H, Ar*H*), 6.23 (t, *J* = 2.9 Hz, 1H, C=C*H*H), 5.68 (t, *J* = 7.3 Hz, 1H, OC*H*), 5.64 (t, *J* = 2.5 Hz, 1H, C=CH*H*), 3.33 (ddt, *J* = 17.4, 8.5, 2.5 Hz, 1H, C*H*HC=CH_2_), 3.02–2.93 (m, 1H, CH*H*C=CH_2_); ^13^C-NMR (125 MHz, CDCl_3_): δ 163.38, 147.52, 133.47, 132.59, 130.68, 129.48, 127.05, 126.81, 23.12, 122.85, 120.68, 117.59, 115.82, 77.30, 77.04, 76.79, 75.28, 73.86, 35.02. HR-MS (ESI): *m*/*z* calcd for C_19_H_13_NNaO_4_ ([M + Na]^+^) 342.0736, found 342.0741.

*4-(2-Benzoyloxyphenyl)-2-methylenebutyrolactone* (**5g**) White solid; mp: 166.3–167.0 °C; 57% yield; ^1^H-NMR (500 MHz, CDCl_3_): δ 8.22 (d, *J* = 7.3 Hz, 2H, Ar*H*), 7.71 (t, *J* = 7.5 Hz, 1H, Ar*H*), 7.57 (t, *J* = 7.8 Hz, 2H, Ar*H*), 7.49 (t, *J* = 7.4 Hz, 1H, Ar*H*), 7.47–7.42 (m, 1H, Ar*H*), 7.35 (dd, *J* = 11.1, 4.0 Hz, 1H, Ar*H*), 7.25 (d, *J* = 8.1 Hz, 1H, Ar*H*), 6.24 (t, *J* = 2.8 Hz, 1H, C=C*H*H), 5.73 (dd, *J* = 8.2, 6.3 Hz, 1H, OC*H*), 5.63 (t, *J* = 2.4 Hz, 1H, C=CH*H*), 3.34 (ddt, *J* = 17.4, 8.4, 2.5 Hz, 1H, CHHC=CH_2_), 2.92 (ddt, *J* = 17.4, 5.9, 2.8 Hz, 1H, CHHC=CH_2_); ^13^C-NMR (125 MHz, CDCl_3_): δ 133.76, 130.21, 128.64, 126.72, 122.12, 78.70, 77.28, 77.03, 76.77, 40.04, 36.41, 29.72, 15.00. HR-MS (ESI): *m*/*z* calcd for C_18_H_1__5_O_4_ ([M + H]^+^) 295.0964, found 295.0964.

*4-[2-(2-Methylbenzoyloxy)phenyl]-2-methylenebutyrolactone* (**5h**) White crystals; mp: 172.8–173.4 °C; 47% yield; ^1^H-NMR (400 MHz, CDCl_3_): δ 8.21–8.12 (m, 1H, Ar*H*), 7.55–7.49 (m, 1H, Ar*H*), 7.49–7.39 (m, 2H, Ar*H*), 7.38–7.28 (m, 3H, Ar*H*), 7.26–7.19 (m, 1H, Ar*H*), 6.22 (t, *J* = 2.9 Hz, 1H, C=C*H*H), 5.70 (dd, *J* = 8.3, 6.1 Hz, 1H, OC*H*), 5.61 (t, *J* = 2.5 Hz, 1H, C=CH*H*), 3.32 (ddt, *J* = 17.4, 8.4, 2.6 Hz, 1H, C*H*HC=CH_2_), 2.90 (ddt, *J* = 17.4, 5.9, 2.9 Hz, 1H, CH*H*C=CH_2_), 2.68 (s, 3H, ArC*H*_3_); ^13^C-NMR (125 MHz, CDCl_3_): δ 170.05, 165.29, 147.67, 141.92, 133.66, 133.33, 132.55, 131.16, 129.52, 127.60, 126.52, 126.17, 122.96, 77.40, 77.08, 76.77, 73.66, 35.30, 22.08.HR-MS (ESI): *m*/*z* calcd for C_19_H_16_NaO_4_ ([M + Na]^+^) 331.0940, found 337.0940.

*4-[2-(3-Methylbenzoyloxy)phenyl]-2-methylenebutyrolactone* (**5i**) White crystals; mp: 179.3–179.9 °C; 45% yield; ^1^H-NMR (500 MHz, CDCl_3_): δ 7.99 (d, *J* = 8.7 Hz, 2H, Ar*H*), 7.48 (t, *J* = 8.0 Hz, 2H, Ar*H*), 7.45–7.39 (m, 2H, Ar*H*), 7.32 (t, *J* = 7.6 Hz, 1H, Ar*H*), 7.21 (d, *J* = 8.0 Hz, 1H, Ar*H*), 6.22 (t, *J* = 2.9 Hz, 1H, C=C*H*H), 5.70 (dd, *J* = 8.3, 6.2 Hz, 1H, OC*H*), 5.60 (t, *J* = 2.5 Hz, 1H, C=CH*H*), 3.31 (ddt, *J* = 17.4, 8.4, 2.5 Hz, 1H, C*H*HC=CH_2_), 2.89 (ddt, *J* = 17.4, 5.9, 2.8 Hz, 1H, CH*H*C=CH_2_), 2.46 (s, 3H, ArCH_3_); ^13^C-NMR (125 MHz, CDCl_3_): δ 170.06, 165.06, 147.69, 138.77, 134.93, 133.65, 132.48, 130.76, 129.52, 128.69, 127.36, 126.56, 126.16, 122.93, 77.30, 77.05, 76.79, 73.66, 35.28, 21.32. HR-MS (ESI): *m*/*z* calcd for C_19_H_16_NaO_4_ ([M + Na]^+^) 337.0940, found 337.0941.

*4-[2-(4-Methylbenzoyloxy)phenyl]-2-methylenebutyrolactone* (**5j**) White crystals; mp: 181.2–181.6 °C; 46% yield; ^1^H-NMR (400 MHz, CDCl_3_): δ 8.08 (d, *J* = 8.2 Hz, 2H, Ar*H*), 7.49–7.38 (m, 2H, Ar*H*), 7.36–7.28 (m, 3H, Ar*H*), 7.24–7.19 (m, 1H, Ar*H*), 6.22 (t, *J* = 2.9 Hz, 1H, C=C*H*H), 5.70 (dd, *J* = 8.3, 6.1 Hz, 1H, OC*H*), 5.60 (t, *J* = 2.5 Hz, 1H, C=CH*H*), 3.31 (ddt, *J* = 17.4, 8.4, 2.6 Hz, 1H, C*H*HC=CH_2_), 2.89 (ddt, *J* = 17.4, 5.9, 2.9 Hz, 1H, CH*H*C=CH_2_), 2.47 (s, 3H, ArCH_3_); ^13^C-NMR (125 MHz, CDCl_3_): δ 170.26, 163.06, 149.69,145.15, 138.77, 133.65, 132.49, 130.29, 129.53, 128.49, 126.51, 126.05, 122.95, 77.35, 77.03, 76.72, 73.71, 35.28, 21.83. HR-MS (ESI): *m*/*z* calcd for C_19_H_16_NaO_4_ ([M + Na]^+^) 331.0940, found 331.0937.

*4-[2-(2-Methoxylbenzoyloxy)phenyl]-2-methylenebutyrolactone* (**5k**) White crystals; mp: 177.8–178.6 °C; 51% yield; ^1^H-NMR (500 MHz, CDCl_3_): δ 8.00 (dd, *J* = 7.7, 1.6 Hz, 1H, Ar*H*), 7.59 (td, *J* = 8.5, 1.7 Hz, 1H, Ar*H*), 7.41 (ddd, *J* = 15.7, 9.2, 4.6 Hz, 2H, Ar*H*), 7.33–7.19 (m, 2H, Ar*H*), 7.07 (dd, *J* = 12.2, 5.2 Hz, 2H, Ar*H*), 6.24 (t, *J* = 2.9 Hz, 1H, C=C*H*H), 5.79 (dd, *J* = 8.2, 6.2 Hz, 1H, OC*H*), 5.61 (t, *J* = 2.5 Hz, 1H, C=CH*H*), 3.95 (s, 3H, ArOC*H*_3_), 3.36 (ddt, *J* = 17.4, 8.3, 2.5 Hz, 1H, C*H*HC=CH_2_), 2.87 (ddt, *J* = 17.4, 5.9, 2.9 Hz, 1H, CH*H*C=CH_2_); ^13^C-NMR (125 MHz, CDCl_3_): δ 170.21, 164.40, 159.83, 147.61, 134.84, 133.89, 132.54, 129.32, 126.40, 125.81, 122.97, 122.72, 120.46, 118.38, 112.25, 77.31, 77.06, 76.80, 73.71, 56.01, 35.45. HR-MS (ESI): *m*/*z* calcd for C_19_H_16_NaO_5_ ([M + Na]^+^) 347.0889, found 347.0889.

*4-[2-(3-Methoxylbenzoyloxy)phenyl]-2-methylenebutyrolactone* (**5l**) White crystals; mp: 180.3–180.8 °C; 50% yield; ^1^H-NMR (500 MHz, CDCl_3_): δ 7.81 (d, *J* = 7.7 Hz, 1H, Ar*H*), 7.76–7.65 (m, 1H, Ar*H*), 7.55–7.40 (m, 3H, Ar*H*), 7.35 (t, *J* = 7.2 Hz, 1H, Ar*H*), 7.30–7.22 (m, 2H, Ar*H*), 6.25 (t, *J* = 2.9 Hz, 1H, C=C*H*H), 5.72 (dd, *J* = 8.3, 6.2 Hz, 1H, OC*H*), 5.63 (t, *J* = 2.5 Hz, 1H, C=CH*H*), 3.92 (s, 3H, ArOC*H*_3_), 3.42–3.22 (m, 1H, C*H*HC=CH_2_), 3.02–2.76 (m, 1H, CH*H*C=CH_2_); ^13^C-NMR (125 MHz, CDCl_3_): δ 169.99, 164.76, 159.88, 147.70, 133.63, 132.44, 129.86, 129.54, 126.60, 126.24, 122.92, 122.56, 120.63, 114.65, 77.28, 77.02, 76.77, 73.69, 55.57, 35.24. HR-MS (ESI): *m*/*z* calcd for C_19_H_16_NaO_5_ ([M + Na]^+^) 347.0889, found 347.0889.

*4-[2-(Butyryloxy)phenyl]-2-methylenebutyrolactone * (**5m**) Colourless oil; 78% yield; ^1^H-NMR (500 MHz, CDCl_3_): δ 7.41–7.33 (m, 2H, Ar*H*), 7.30–7.21 (m, 1H, Ar*H*), 7.16–7.05 (m, 1H, Ar*H*), 6.31 (t, *J* = 2.8 Hz, 1H, C=C*H*H), 5.68 (t, *J* = 2.4 Hz, 1H, C=CH*H*), 5.61 (dd, *J* = 8.3, 6.3 Hz, 1H, OC*H*), 3.34 (ddt, *J* = 17.4, 8.4, 2.5 Hz, 1H, C*H*HC=CH_2_), 2.86 (ddt, *J* = 17.4, 5.9, 2.8 Hz, 1H, CH*H*C=CH_2_), 2.61–2.50 (m, 2H, CH_3_CH_2_CH_2_), 1.78 (dt, *J* = 14.8, 7.4 Hz, 2H, CH_3_CH_2_CH_2_), 1.05 (dd, *J* = 9.0, 5.9 Hz, 3H, CH_3_CH_2_CH_2_); ^13^C-NMR (125 MHz, CDCl_3_): δ 171.75, 147.64, 133.87, 131.95, 129.47, 126.31, 77.34, 77.08, 76.83, 73.94, 36.12, 35.23, 18.41, 13.68. HR-MS (ESI): *m*/*z* calcd for C_1__5_H_16_NaO_4_ ([M + Na]^+^) 283.0840, found 283.0841.

*4-[2-(Cinnamoyloxy)phenyl]-2-methylenebutyrolactone* (**5n**) Yellow oil; 48% yield; ^1^H-NMR (500 MHz, CDCl_3_): δ 7.84 (d, *J* = 15.9 Hz, 1H, Ar*H*), 7.49–7.39 (m, 2H, Ar*H*), 7.32 (t, *J* = 7.5 Hz, 1H, Ar*H*), 7.29 (s, 1H, Ar*H*), 7.24–7.17 (m, 2H, Ar*H*), 7.01–6.97 (m, 1H, Ar*H*), 6.48 (d, *J* = 15.9 Hz, 1H, Ar*H*), 6.30 (t, *J* = 2.7 Hz, 1H, C=C*H*H), 5.71 (dd, *J* = 8.2, 6.4 Hz, 1H, OC*H*), 5.66 (d, *J* = 7.3 Hz, 1H, C=CH*H*), 4.00 (s, 2H, CH=CH), 3.39 (dd, *J* = 17.5, 8.5 Hz, 1H, C*H*HC=CH_2_), 2.99–2.84 (m, 1H, CH*H*C=CH_2_); ^13^C-NMR (125 MHz, CDCl_3_): δ 148.74, 147.72, 129.49, 127.04, 126.21, 123.67, 122.95, 122.72, 114.93, 113.54, 109.70, 77.27, 77.01, 76.76, 74.01, 56.04, 35.16, 26.33. HR-MS (ESI): *m*/*z* calcd for C_21_H_1__9_O_6_ ([M + H]^+^) 367.1173, found 317.1176.

*4-[3-(3-Chlorobenzoyloxy)phenyl]-2-methylenebutyrolactone* (**6a**) White solid; mp: 205.7–206.4 °C; 52% yield; ^1^H-NMR (500 MHz, CDCl_3_): δ 8.20 (s, 1H, Ar*H*), 8.10 (d, *J* = 7.7 Hz, 1H, Ar*H*), 7.65 (dd, *J* = 8.0, 1.0 Hz, 1H, Ar*H*), 7.55–7.45 (m, 2H, Ar*H*), 7.30–7.19 (m, 3H, Ar*H*), 6.35 (t, *J* = 2.8 Hz, 1H, C=C*H*H), 5.74 (t, *J* = 2.4 Hz, 1H, C=CH*H*), 5.62–5.54 (m, 1H, OC*H*), 3.52–3.41 (m, 1H, C*H*HC=CH_2_), 2.97 (ddt, *J* = 12.2, 6.0, 2.9 Hz, 1H, CH*H*C=CH_2_); ^13^C-NMR (125 MHz, CDCl_3_): δ 163.87, 151.10, 141.83, 134.86, 133.76, 131.04, 130.35, 129.90, 128.31, 122.93, 121.78, 118.66, 77.41, 76.88, 76.76, 36.21, 29.70. HR-MS (ESI): *m*/*z* calcd for C_18_H_13_ClNaO_4_ ([M + Na]^+^) 351.0394, found 351.0395.

*4-[3-(4-Chlorobenzoyloxy)phenyl]-2-methylenebutyrolactone* (**6b**) White solid; mp: 209.8–210.5 °C; 64% yield; ^1^H-NMR (400 MHz, CDCl_3_): δ 8.22–8.03 (m, 2H, Ar*H*), 7.61–7.36 (m, 3H, Ar*H*), 7.33–7.14 (m, 3H, Ar*H*), 6.32 (t, *J* = 2.8 Hz, 1H, C=C*H*H), 5.71 (t, *J* = 2.5 Hz, 1H, C=CH*H*), 5.56 (dd, *J* = 7.8, 6.7 Hz, 1H, OC*H*), 3.44 (ddt, *J* = 17.1, 8.1, 2.5 Hz, 1H, C*H*HC=CH_2_), 2.94 (ddt, *J* = 17.1, 6.2, 2.9 Hz, 1H, CH*H*C=CH_2_); ^13^C-NMR (125 MHz, CDCl_3_): δ 164.24, 151.14, 141.80, 140.37, 133.73, 131.58, 130.12, 129.05, 127.72, 122.93, 121.85, 118.72, 77.52 ,76.91, 76.74, 36.21. HR-MS (ESI): *m*/*z* calcd for C_18_H_13_ClNaO_4_ ([M + Na]^+^) 351.0394, found 351.0394.

*4-[3-(2-Bromobenzoyloxy)phenyl]-2-methylenebutyrolactone* (**6c**) White crystals; mp: 175.3–175.8 °C; 56% yield; ^1^H-NMR (500 MHz, CDCl_3_): δ 8.06 (dd, *J* = 7.6, 1.7 Hz, 1H, Ar*H*), 7.83–7.72 (m, 1H, Ar*H*), 7.61–7.42 (m, 2H, Ar*H*), 7.30 (d, *J* = 10.2 Hz, 4H, Ar*H*), 6.38 (t, *J* = 2.8 Hz, 1H, C=C*H*H), 5.76 (dd, *J* = 6.3, 3.9 Hz, 1H, C=CH*H*), 5.67–5.40 (m, 1H, OC*H*), 3.62–3.30 (m, 1H, C*H*HC=CH_2_), 3.05–2.88 (m, 1H, CH*H*C=CH_2_); ^13^C-NMR (125 MHz, CDCl_3_): δ 134.74, 133.35, 130.12, 127.40, 126.70, 122.91, 118.69, 77.28, 77.02, 76.77, 36.35, 29.71, 19.20. HR-MS (ESI): *m*/*z* calcd for C_18_H_13_BrNaO_4_ ([M + Na]^+^) 394.9889, found 394.9892.

*4-[3-(3-Bromobenzoyloxy)phenyl]-2-methylenebutyrolactone* (**6d**) White crystals; mp: 178.6–178.9 °C; 63% yield; ^1^H-NMR (500 MHz, CDCl_3_): δ 8.31 (d, *J* = 52.5 Hz, 1H, Ar*H*), 8.10 (dd, *J* = 52.3, 7.5 Hz, 1H, Ar*H*), 7.84–7.72 (m, 1H, Ar*H*), 7.54–7.34 (m, 2H, Ar*H*), 7.33–7.17 (m, 3H, Ar*H*), 6.36 (t, *J* = 2.8 Hz, 1H, C=C*H*H), 5.74 (t, *J* = 2.5 Hz, 1H, C=CH*H*), 5.66–5.46 (m, 1H, OC*H*), 3.60–3.36 (m, 1H, C*H*HC=CH_2_), 2.97 (ddt, *J* = 17.1, 6.1, 2.9 Hz, 1H, CH*H*C=CH_2_); ^13^C-NMR (125 MHz, CDCl_3_): δ 136.72, 133.14, 132.05, 131.69, 130.17, 128.76, 126.91, 126.53, 122.94, 121.78, 118.66, 77.37, 76.87, 76.76, 36.21, 29.72, 15.00. HR-MS (ESI): *m*/*z* calcd for C_18_H_13_BrNaO_4_ ([M + Na]^+^) 394.9894, found 394.9890.

*4-[3-(4-Bromobenzoyloxy)phenyl]-2-methylenebutyrolactone* (**6e**) White crystals; mp: 168.6–169.4 °C; 41% yield; ^1^H-NMR (500 MHz, CDCl_3_): δ 8.10 (d, *J* = 8.5 Hz, 2H, Ar*H*), 8.00 (d, *J* = 8.4 Hz, 2H, Ar*H*), 7.69 (dd, *J* = 24.8, 8.4 Hz, 4H, Ar*H*), 6.37 (t, *J* = 2.8 Hz, 1H, C=C*H*H), 5.76 (t, *J* = 2.4 Hz, 1H, C=CH*H*), 5.61 (s, 1H, OC*H*), 4.35 (t, *J* = 6.8 Hz, 1H, C*H*HC=CH_2_), 3.48 (dd, *J* = 17.0, 8.1 Hz, 1H, CH*H*C=CH_2_). ^13^C-NMR (125 MHz, CDCl_3_): δ 150.78, 137.95, 133.05, 131.69, 129.05, 127.28, 126.76, 122.75, 122.08, 78.62, 77.28, 77.03, 76.78, 40.03, 36.36, 15.00. HR-MS (ESI): *m*/*z* calcd for C_18_H_13_BrNaO_4_ ([M + Na]^+^) 394.9890, found 394.9889.

*4-(3-Benzoyloxyphenyl)-2-methylenebutyrolactone* (**6f**) White solid; mp: 204.5–204.8 °C; 52% yield; ^1^H-NMR (500 MHz, CDCl_3_): δ 8.22 (d, *J* = 7.3 Hz, 2H, Ar*H*), 7.68 (t, *J* = 7.5 Hz, 1H, Ar*H*), 7.49 (t, *J* = 7.8 Hz, 1H, Ar*H*), 7.32–7.20 (m, 5H, Ar*H*), 6.35 (t, *J* = 2.8 Hz, 1H, C=C*H*H), 5.73 (t, *J* = 2.4 Hz, 1H, C=CH*H*), 5.62–5.52 (m, 1H, OC*H*), 3.46 (ddt, *J* = 17.1, 8.1, 2.4 Hz, 1H, C*H*HC=CH_2_), 2.98 (ddt, *J* = 9.2, 6.0, 2.8 Hz, 1H, CH*H*C=CH_2_); ^13^C-NMR (125 MHz, CDCl_3_): δ 135.76, 131.21, 128.69, 126.77, 122.62, 78.70, 77.28, 77.03, 76.77, 42.04, 36.51, 29.79, 15.20. HR-MS (ESI): *m*/*z* calcd for C_18_H_14_NaO_4_ ([M + Na]^+^) 317.0785, found 317.0784.

*4-[3-(2-Methylbenzoyloxy)phenyl]-2-methylenebutyrolactone* (**6g**) White crystals; mp: 178.3–178.9 °C; 57% yield; ^1^H-NMR (500 MHz, CDCl_3_): δ 8.18 (d, *J* = 7.8 Hz, 1H, Ar*H*), 7.54–7.46 (m, 2H, Ar*H*), 7.36 (t, *J* = 7.7 Hz, 2H, Ar*H*), 7.24 (dd, *J* = 13.8, 7.7 Hz, 3H, Ar*H*), 6.35 (t, *J* = 2.8 Hz, 1H, C=C*H*H), 5.73 (t, *J* = 2.4 Hz, 1H, C=CH*H*), 5.64–5.51 (m, 1H, OC*H*), 3.46 (ddt, *J* = 17.1, 8.1, 2.4 Hz, 1H, C*H*HC=CH_2_), 2.98 (ddt, *J* = 17.1, 6.1, 2.9 Hz, 1H, CH*H*C=CH_2_), 2.70 (s, 3H, ArCH_3_); ^13^C-NMR (125 MHz, CDCl_3_): δ 165.62, 151.30, 141.68, 141.44, 133.81, 132.91, 132.02, 131.21, 130.02, 128.21, 125.98, 122.71, 122.06, 118.92, 77.26, 77.01, 76.76, 36.23, 21.96. HR-MS (ESI): *m*/*z* calcd for C_19_H_16_NaO_4_ ([M + Na]^+^) 331.0940, found 331.0940.

*4-[3-(3-Methylbenzoyloxy)phenyl]-2-methylenebutyrolactone* (**6h**) White crystals; mp: 177.6–178.2 °C; 68% yield; ^1^H-NMR (500 MHz, CDCl_3_): δ 8.03 (s, 1H, Ar*H*), 7.46 (ddd, *J* = 15.1, 9.9, 5.9 Hz, 3H, Ar*H*), 7.28 (d, *J* = 9.6 Hz, 4H, Ar*H*), 6.35 (t, *J* = 2.8 Hz, 1H, C=C*H*H), 5.73 (t, *J* = 2.4 Hz, 1H, C=CH*H*), 5.66–5.52 (m, 1H, OC*H*), 3.50–3.39 (m, 1H, C*H*HC=CH_2_), 3.03–2.93 (m, 1H, CH*H*C=CH_2_), 2.48 (s, 3H, ArCH_3_); ^13^C-NMR (125 MHz, CDCl_3_): δ 170.05, 165.28, 152.07, 136.51, 137.36, 134.45, 134.61, 130.71, 129.69, 128.53, 127.37, 126.67, 122.26, 77.48, 77.28, 77.02, 76.77, 36.32, 21.30. HR-MS (ESI): *m*/*z* calcd for C_19_H_16_NaO_4_ ([M + Na]^+^) 331.0942, found 331.0940.

*4-[3-(4-Methylbenzoyloxy)phenyl]-2-methylenebutyrolactone* (**6i**) White crystals; mp: 168.5–168.9 °C; 57% yield; ^1^H-NMR (500 MHz, CDCl_3_): δ 8.10 (d, *J* = 8.1 Hz, 2H, Ar*H*), 7.48 (t, *J* = 7.8 Hz, 1H, Ar*H*), 7.34 (d, *J* = 8.0 Hz, 1H, Ar*H*), 7.30–7.20 (m, 4H, Ar*H*), 6.35 (t, *J* = 2.8 Hz, 1H, C=C*H*H), 5.73 (t, *J* = 2.5 Hz, 1H, C=CH*H*), 5.70–5.40 (m, 1H, OC*H*), 3.56–3.36 (m, 1H, C*H*HC=CH_2_), 2.98 (ddt, *J* = 17.1, 6.0, 2.9 Hz, 1H, CH*H*C=CH_2_), 2.34 (d, *J* = 144.3 Hz, 3H, ArCH_3_). ^13^C-NMR (125 MHz, CDCl_3_): δ 169.99, 165.13, 151.11, 144.66, 141.64, 130.24, 130.01, 129.36, 127.37, 126.58, 122.79, 122.63, 122.00, 118.87, 77.27, 77.01, 76.76, 36.22, 21.77. HR-MS (ESI): *m*/*z* calcd for C_19_H_16_NaO_4_ ([M + Na]^+^) 331.0940, found 331.0941.

*4-[3-(2-Methoxylbenzoyloxy)phenyl]-2-methylenebutyrolactone* (**6j**) White crystals; mp: 172.3–172.8 °C; 45% yield; ^1^H-NMR (500 MHz, CDCl_3_): δ 8.07–7.99 (m, 1H, Ar*H*), 7.61–7.56 (m, 1H, Ar*H*), 7.32–7.20 (m, 4H, Ar*H*), 7.08 (t, *J* = 7.6 Hz, 2H, Ar*H*), 6.34 (t, *J* = 2.8 Hz, 1H, C=C*H*H), 5.73 (t, *J* = 2.4 Hz, 1H, C=CH*H*), 5.62–5.43 (m, 1H, OC*H*), 3.97 (s, 3H, ArOC*H*_3_), 3.47–3.38 (m, 1H, C*H*HC=CH_2_), 2.97 (ddt, *J* = 17.1, 6.1, 2.9 Hz, 1H, CH*H*C=CH_2_). ^13^C-NMR (125 MHz, CDCl_3_): δ 151.37, 141.52, 134.54, 133.87, 132.27, 129.92, 122.76, 122.54, 122.09, 120.27, 118.98, 112.26, 77.30, 77.02, 76.76, 56.07, 49.16, 36.23, 33.95, 25.63, 24.94. HR-MS (ESI): *m*/*z* calcd for C_19_H_16_NaO_5_ ([M + Na]^+^) 347.0889, found 347.0890.

*4-[3-(3-Methoxybenzoyloxy)phenyl]-2-methylenebutyrolactone* (**6k**) White crystals; mp: 183.7–184.2 °C; 57% yield; ^1^H-NMR (500 MHz, CDCl_3_): δ 7.82 (d, *J* = 7.7 Hz, 1H, Ar*H*), 7.73 (d, *J* = 7.7 Hz, 1H, Ar*H*), 7.47 (dt, *J* = 18.6, 7.8 Hz, 2H, Ar*H*), 7.31–7.19 (m, 4H, Ar*H*), 6.35 (t, *J* = 2.8 Hz, 1H, C=C*H*H), 5.73 (t, *J* = 2.4 Hz, 1H, C=CH*H*), 5.62–5.55 (m, 1H, OC*H*), 3.92 (s, 3H, ArOC*H*_3_), 3.46 (ddt, *J* = 17.0, 8.1, 2.4 Hz, 1H, C*H*HC=CH_2_), 3.01–2.91 (m, 1H, CH*H*C=CH_2_). ^13^C-NMR (125 MHz, CDCl_3_): δ 159.75, 151.34, 141.70, 133.78, 130.05, 129.67, 129.49, 122.95–122.49, 121.94, 120.39, 118.80, 114.51, 77.27, 77.01, 76.76, 55.49, 36.21, 29.70.HR-MS (ESI): *m*/*z* calcd for C_19_H_16_NaO_5_ ([M + Na]^+^) 347.0889, found 347.0889.

*4-[3-(4-Methoxybenzoyloxy)phenyl]-2-methylenebutyrolactone* (**6l**) White crystals; mp: 182.6–183.3 °C; 56% yield; ^1^H-NMR (500 MHz, CDCl_3_): δ 8.23–8.07 (m, 2H, Ar*H*), 7.45 (t, *J* = 8.0 Hz, 1H, Ar*H*), 7.29–7.14 (m, 3H, Ar*H*), 7.02–6.92 (m, 2H, Ar*H*), 6.32 (t, *J* = 2.8 Hz, 1H, C=C*H*H), 5.70 (t, *J* = 2.5 Hz, 1H, C=CH*H*), 5.55 (dd, *J* = 7.8, 6.8 Hz, 1H, OC*H*), 3.90 (s, 3H, ArOC*H*_3_), 3.43 (ddt, *J* = 17.1, 8.1, 2.5 Hz, 1H, C*H*HC=CH_2_), 3.00–2.87 (m, 1H, CH*H*C=CH_2_); ^13^C-NMR (125 MHz, CDCl_3_): δ 169.99, 164.80, 164.06, 151.44, 141.61, 133.83, 132.35, 130.00, 122.84, 122.60, 122.06, 121.52, 118.92, 113.93, 77.35, 77.06, 76.74, 55.56, 36.21.HR-MS (ESI): *m*/*z* calcd for C_19_H_16_NaO_5_ ([M + Na]^+^) 347.0889, found 347.0892.

*4-(3-Propionyloxyphenyl)-2-methylenebutyrolactone* (**6m**) Colourless oil; 56% yield; ^1^H-NMR (500 MHz, CDCl_3_): δ 7.42 (t, *J* = 8.3 Hz, 1H, Ar*H*), 7.29 (s, 1H, Ar*H*), 7.20 (d, *J* = 7.8 Hz, 1H, Ar*H*), 7.09 (s, 1H, Ar*H*), 6.34 (t, *J* = 2.8 Hz, 1H, C=C*H*H), 5.72 (t, *J* = 2.4 Hz, 1H, C=CH*H*), 5.64–5.43 (m, 1H, OC*H*), 3.44 (ddt, *J* = 17.1, 8.1, 2.4 Hz, 1H, C*H*HC=CH_2_), 2.94 (ddt, *J* = 17.1, 6.0, 2.8 Hz, 1H, CH*H*C=CH_2_), 2.62 (q, *J* = 7.5 Hz, 2H, CH_3_CH_2_), 1.29 (t, *J* = 7.5 Hz, 3H, CH_3_CH_2_); ^13^C-NMR (125 MHz, CDCl_3_): δ 172.81, 151.14, 141.57, 133.78, 129.92, 122.78, 122.53, 121.75, 118.63, 77.23, 77.01, 76.75, 36.20, 27.73, 9.02. HR-MS (ESI): *m*/*z* calcd for C_14_H_14_NaO_4_ ([M + Na]^+^) 269.0784, found 269.0784.

*4-(3-Cinnamoyloxyphenyl)-2-methylenebutyrolactone* (**6n**) Yellow oil; 34% yield; ^1^H-NMR (400 MHz, CDCl_3_): δ 7.87 (d, *J* = 16.0 Hz, 1H, Ar*H*), 7.64–7.55 (m, 2H, CH=CH), 7.47–7.38 (m, 4H, Ar*H*), 7.25–7.10 (m, 3H, Ar*H*), 6.62 (d, *J* = 16.0 Hz, 1H, Ar*H*), 6.31 (t, *J* = 2.8 Hz, 1H, C=C*H*H), 5.70 (t, *J* = 2.5 Hz, 1H, C=CH*H*), 5.60–5.45 (m, 1H, OC*H*), 3.42 (ddt, *J* = 17.1, 8.1, 2.5 Hz, 1H, C*H*HC=CH_2_), 2.93 (ddt, *J* = 17.1, 6.0, 2.9 Hz, 1H, CH*H*C=CH_2_); ^13^C-NMR (100 MHz, CDCl_3_): δ 169.99, 165.26, 151.17, 147.00, 141.64, 134.07, 133.83, 130.88, 130.01, 129.06, 128.38, 122.76, 121.88, 118.76, 116.98, 77.56,76.95, 76.78, 36.19. HR-MS (ESI): *m*/*z* calcd for C_20_H_16_NaO_4_ ([M + Na]^+^) 343.0940, found 343.0941.

#### 3.2.3. General Synthetic Procedure for Ether Compounds

K_2_CO_3_ (13.8 mg, 10.0 mmol) and Cs_2_CO_3_ (3.2 mg, 1.0 mmol) as catalyst, intermediate compounds **5** or **6** (196.0 mg, 1.1 mmol), and acetonitrile (35.0 mL) were added into a round bottomed flask and refluxed at 78 °C. Then, the appropriate brominated alkane (1.2 mmol) was slowly added into the mixture and stirred for 12 h at 78 °C. The progress of the reaction was monitored by TLC. After complete conversion, the suspension was filtered and washed with acetonitrile. Finally, the solution was dried with anhydrous Na_2_SO_4_, filtered, and evaporated under vacuum. The obtained crude products were also purified by column chromatography. The ^1^H-NMR, ^13^C-NMR, and HR-ESI-MS data are listed below.

*4-(2-Butoxyphenyl)-2-methylenebutyrolactone* (**5o**) Yellow oil; 42% yield; ^1^H-NMR (500 MHz, CDCl_3_): δ 7.38–7.30 (m, 2H, Ar*H*), 7.04–6.89 (m, 2H, Ar*H*), 6.33 (t, *J* = 2.8 Hz, 1H, C=C*H*H), 5.77 (dd, *J* = 8.4, 6.0 Hz, 1H, OC*H*), 5.67 (t, *J* = 2.4 Hz, 1H, C=CH*H*), 4.13–3.95 (m, 2H, CH_3_CH_2_CH_2_CH_2_O), 3.45 (ddt, *J* = 17.4, 8.5, 2.6 Hz, 1H, C*H*HC=CH_2_), 2.92 (ddt, *J* = 17.4, 5.7, 2.8 Hz, 1H, CH*H*C=CH_2_), 1.86–1.76 (m, 2H, CH_3_CH_2_CH_2_CH_2_O), 1.57–1.47 (m, 2H, CH_3_CH_2_CH_2_CH_2_O), 1.05–0.98 (m, 3H, CH_3_CH_2_CH_2_CH_2_O); ^13^C-NMR (100 MHz, CDCl_3_): δ 155.78, 134.92, 129.42, 126.12, 121.82, 120.40, 111.27, 77.29, 76.78, 75.21, 67.79, 35.04, 31.28, 30.92, 19.41, 13.83. HR-MS (ESI): *m*/*z* calcd for C_15_H_18_NaO_3_ ([M + Na]^+^) 269.1148, found 269.1147.

*4-(2-Propoxylphenyl)-2-methylenebutyrolactone* (**5p**) Yellow oil; 38% yield; ^1^H-NMR (500 MHz, CDCl_3_): δ 7.30 (dd, *J* = 13.1, 5.4 Hz, 1H, Ar*H*), 6.90 (dd, *J* = 12.5, 10.8 Hz, 3H, Ar*H*), 6.33 (t, *J* = 2.8 Hz, 1H, C=C*H*H), 5.71 (t, *J* = 2.4 Hz, 1H, C=CH*H*), 5.58–5.41 (m, 1H, OC*H*), 3.94 (t, *J* = 6.5 Hz, 2H, CH_3_CH_2_CH_2_O), 3.41 (ddt, *J* = 17.1, 8.1, 2.4 Hz, 1H, C*H*HC=CH_2_), 2.93 (ddt, *J* = 17.1, 6.1, 2.9 Hz, 1H, CH*H*C=CH_2_), 1.90–1.78 (m, 2H, CH_3_CH_2_CH_2_O), 1.12–0.97 (m, 3H, CH_3_CH_2_CH_2_O); ^13^C-NMR (125 MHz, CDCl_3_): δ 170.14, 159.58, 141.41, 134.20, 129.93, 122.43, 117.30, 114.49, 111.51, 77.82, 77.28, 77.03, 76.77, 69.61, 22.57, 10.51. HR-MS (ESI): *m*/*z* calcd for C_14_H_16_NaO_3_ ([M + Na]^+^) 255.0991, found 255.0992.

*4-(2-Isopropoxylphenyl)-2-methylenebutyrolactone* (**5q**) Yellow oil; 47% yield; ^1^H-NMR (500 MHz, CDCl_3_): δ 7.33 (dd, *J* = 12.7, 6.6 Hz, 2H, Ar*H*), 6.96 (dt, *J* = 12.8, 5.9 Hz, 2H, Ar*H*), 6.32 (t, *J* = 2.9 Hz, 1H, C=C*H*H), 5.73 (dd, *J* = 8.4, 5.9 Hz, 1H, OC*H*), 5.67 (t, *J* = 2.4 Hz, 1H, C=CH*H*), 4.67 (dt, *J* = 12.1, 6.0 Hz, 1H, (CH_3_)_2_CHO), 3.45 (ddt, *J* = 17.4, 8.5, 2.6 Hz, 1H, C*H*HC=CH_2_), 2.91 (ddt, *J* = 17.5, 5.7, 2.8 Hz, 1H, CH*H*C=CH_2_), 1.38 (dd, *J* = 8.8, 6.1 Hz, 6H, (CH_3_)_2_CHO); ^13^C-NMR (125 MHz, CDCl_3_): δ 170.74, 154.58, 135.17, 129.35, 129.06, 126.55, 121.63, 120.19, 112.42, 77.29, 77.04, 76.78, 75.52, 69.95, 34.95, 29.72, 22.01. HR-MS (ESI): *m*/*z* calcd for C_14_H_16_NaO_3_ ([M + Na]^+^) 255.0991, found 255.0990.

*4-(2-Ethoxyphenyl)-2-methylenebutyrolactone* (**5r**) Yellow oil; 46% yield; ^1^H-NMR (400 MHz, CDCl_3_): δ 7.31–7.25 (m, 3H, Ar*H*), 6.95 (td, *J* = 7.5, 0.8 Hz, 1H, Ar*H*), 6.28 (t, *J* = 2.9 Hz, 1H, C=C*H*H), 5.71 (dd, *J* = 8.5, 5.8 Hz, 1H, OC*H*), 5.63 (t, *J* = 2.5 Hz, 1H, C=CH*H*), 4.06 (qd, *J* = 7.0, 2.6 Hz, 2H, CH_3_CH_2_O), 3.42 (dd, *J* = 17.4, 8.5 Hz, 1H, C*H*HC=CH_2_), 2.95–2.77 (m, 1H, CH*H*C=CH_2_), 1.40 (t, *J* = 7.0 Hz, 3H, CH_3_CH_2_O).^13^C-NMR (125 MHz, CDCl_3_): δ 129.48, 126.34, 121.70, 120.42, 111.34, 77.29, 77.03, 76.71, 75.44, 63.64, 34.90, 14.73. HR-MS (ESI): *m*/*z* calcd for C_13_H_14_NaO_3_ ([M + Na]^+^) 241.0835, found 241.0834.

*4-(3-Butoxyphenyl)-2-methylenebutyrolactone* (**6o**) Yellow oil; 47% yield; ^1^H-NMR (500 MHz, CDCl_3_): δ 7.32 (dd, *J* = 13.2, 5.4 Hz, 2H, Ar*H*), 6.91 (d, *J* = 9.0 Hz, 2H, Ar*H*), 6.35 (t, *J* = 2.8 Hz, 1H, C=C*H*H), 5.73 (t, *J* = 2.4 Hz, 1H, C=CH*H*), 5.63–5.40 (m, 1H, OC*H*), 4.01 (t, *J* = 6.5 Hz, 2H, CH_3_CH_2_ CH_2_CH_2_O), 3.43 (ddt, *J* = 17.0, 8.0, 2.4 Hz, 1H, C*H*HC=CH_2_), 2.95 (ddt, *J* = 17.1, 6.1, 2.9 Hz, 1H, CH*H*C=CH_2_), 1.86–1.74 (m, 2H, CH_3_CH_2_CH_2_CH_2_O), 1.58–1.47 (m, 2H, CH_3_CH_2_CH_2_CH_2_O), 1.09–0.96 (m, 3H, CH_3_CH_2_CH_2_CH_2_O); ^13^C-NMR (100 MHz, CDCl_3_): δ 155.78, 134.92, 129.42, 126.12, 121.82, 120.40, 111.27, 77.29, 76.78, 75.21, 67.79, 35.04, 31.28, 30.92, 19.41, 13.83. HR-MS (ESI): *m*/*z* calcd for C_15_H_18_NaO_3_ ([M + Na]^+^) 269.1148, found 269.1147.

*4-(3-Propoxylphenyl)-2-methylenebutyrolactone* (**6p**) Yellow oil; 54% yield; ^1^H-NMR (500 MHz, CDCl_3_): δ 7.30 (dd, *J* = 13.1, 5.4 Hz, 1H, Ar*H*), 6.90 (dd, *J* = 12.5, 10.8 Hz, 3H, Ar*H*), 6.33 (t, *J* = 2.8 Hz, 1H, C=C*H*H), 5.71 (t, *J* = 2.4 Hz, 1H, C=CH*H*), 5.58–5.41 (m, 1H, OC*H*), 3.94 (t, *J* = 6.5 Hz, 2H, CH_3_CH_2_CH_2_O), 3.41 (ddt, *J* = 17.1, 8.1, 2.4 Hz, 1H, C*H*HC=CH_2_), 2.93 (ddt, *J* = 17.1, 6.1, 2.9 Hz, 1H, CH*H*C=CH_2_), 1.90–1.78 (m, 2H, CH_3_CH_2_CH_2_O), 1.12–0.97 (m, 3H, CH_3_CH_2_CH_2_O); ^13^C-NMR (125 MHz, CDCl_3_): δ 170.14, 159.58, 141.41, 134.20, 129.93, 122.43, 117.30, 114.49, 111.51, 77.82, 77.28, 77.03, 76.77, 69.61, 22.57, 10.51. HR-MS (ESI): *m*/*z* calcd for C_14_H_16_NaO_3_ ([M + Na]^+^) 255.0991, found 255.0992.

### 3.3. Fungicidal Activity Bioassay

#### 3.3.1. Preparation of Spore Suspension

The fungal pathogens *C. lagenarium* and *B. cinerea* was provided by the Agricultural Culture Collection of China (Yangling, Shaanxi, China). *C. lagenarium* was cultured for 2 weeks at 25 ± 1 °C on potato dextrose agar (PDA) while *B. cinerea* was cultured at 20 °C on the same medium after being retrieved from the storage tube. Plates were flooded with sterile distilled water, and then conidia were scraped with a glass rod. Mycelial debris was removed by filtration. The spores were harvested and suspended in sterile distilled water containing 0.1% (*v*/*v*) Tween 20. The concentration of the spore suspension was adjusted to 1.0 × 10^6^ spores/mL with sterilized distilled water following [21,39].

#### 3.3.2. Spore Germination Assay

The tested samples (10.0 mg) dissolved in acetone (0.1 mL) were diluted with sterile distilled water to prepare 10.0 mL stock solution, which was further diluted to prepare test solutions in which the final concentration of acetone was <1% (*v*/*v*). A series of concentrations of tested samples and one control (1% acetone with sterile distilled water) were separately tested for spore germination of *C. lagenarium* or *B. cinerea*. The samples were inoculated with spore suspension of *C. lagenarium* or *B. cinerea* containing 1.0 × 10^6^ spores/mL. Aliquots of 10 μL of prepared spore suspension were placed on separate glass slides in triplicate. Slides containing the spores were incubated in a moisture chamber at 25 °C for 6~8 h. Each slide was then observed under the microscope for spore germination. Spores were considered to have germinated if the length of the germ tube was at least half the length of the spore. Afterward, spore germination was stopped by applying a drop of lactophenol-cotton blue to the inoculation sites on plates. The numbers of generated spores were counted under a microscope (Olympus BX61, Tokyo, Japan), and the percentage of germinated spores was calculated. Chlorothalonil was used as the positive control [21,39].

### 3.4. Building and Validation of the QSAR Model

Firstly, the optimal conformers of the title compounds with the lowest energy were computed at the DFT/6-31G (d) level using the Gaussian 03W package of programs [40]. Then, the calculated results were changed into a form compatible with CODESSA 2.7.15 using Ampac 9.1.3 [41,42]. Finally, all of the molecular descriptors involved in these compounds were calculated by CODESSA 2.7.15. In order to find out which structural features play an important role in the fungicidal activity against *C. lagenarium*, the heuristic method analysis was selected to generate the QSAR model. In this model, the statistical criteria were indicated by the squared correction coefficient (*R*^2^), the squared standard error of the estimates (*S*^2^), and the Fisher significance ratio (*F*). The tested IC_50_ values were converted into the corresponding log IC_50_ values and used as dependent variables in the QSAR studies. The quality of the final model was determined using both an internal validation and the “leave-one-out” cross-validation methods [43].

## 4. Conclusions

In summary, forty-six ester and six ether derivatives containing α-methylene-γ-butyrolactone moieties were synthesized, and their fungicidal activities against *C. lagenarium* and *B. cinerea* was investigated. Halogen atom-containing derivatives showed better activity than others, especially compounds **6a**,**d** which exhibited excellent fungicidal activity against *C. lagenarium*. Both SAR and QSAR studies indicated that the structural characteristics had an important influence on the fungicidal activity, and electron withdrawing substituents on the α-methylene-γ-butyrolactone derivatives had a positive effect on the fungicidal activity. It was notable that the present set of compounds consisted of racemic mixtures; it will be an interesting task for further studies to test the most active compounds in optically pure form. The level of fungicidal activity and cytotoxic activity observed with α-methylene-γ-butyrolactone derivatives provide great impetus for further work on the design of high-activity and non-toxic crop-protection agents.

## References

[1] Shimizu M., Yazawa S., Ushijima Y. (2009). A promising strain of endophytic *Streptomyces* sp. for biological control of cucumber anthracnose. J. Gen. Plant Pathol..

[2] Haverkort A., Boonekamp P., Hutten R., Jacobsen E., Lotz L., Kessel G., Visser R., van der Vossen E. (2008). Societal costs of late blight in potato and prospects of durable resistance through cisgenic modification. Potato Res..

[3] Brase S., Encinas A., Keck J., Nising C.F. (2009). Chemistry and biology of mycotoxins and relatedfungal metabolites. Chem. Rev..

[4] Wilson R.A., Talbot N.J. (2009). Fungal physiology—A future perspective. Microbiology.

[5] Kuan C.P., Wu M.T., Huang H.C., Chang H. (2011). Rapid detection of Colletotrichum lagenarium, causal agent of anthracnose of cucurbitaceous crops, by PCR and real-time PCR. J. Phytopathol..

[6] Williamson B., Tudzynski B., Tudzynski P., van Kan J.A.L. (2007). Botrytis cinerea: The cause of grey mould disease. Mol. Plant Pathol..

[7] Fisher M.C., Henk D.A., Briggs C.J., Brownstein J.S., Madoff L.C., McCraw S.L., Gurr S.J. (2012). Emerging fungal threats to animal, plant and ecosystem health. Nature.

[8] Chen Y., Dai G. (2012). Antifungal activity of plant extracts against *Colletotrichum lagenarium*, the causal agent of anthracnose in cucumber. J. Sci. Food Agric..

[9] Dodds P.N., Rathjen J.P. (2010). Towards an integrated view of plant pathogen interactions. Nat. Rev. Genet..

[10] Han C., Barrios F.J., Mark V.R., David A.C. (2009). Semisynthetic Derivatives of Sesquiterpene Lactones by Palladium-Catalyzed Arylation of the α-Methylene-γ-lactone Substructure. J. Org. Chem..

[11] Ravinder R., Laura J.A., Le T., Christopher D.T., Amy R.H. (2007). Cross Metathesis of α-Methylene Lactones II: γ- and δ-Lactones. Org. Lett..

[12] Yusuke M., Masaki T. (2013). Construction of spiro-Fused 2-oxindole/α-methylene-γ-Butyrolactone Systems with extremely high enantioselectivity via Indium-Catalyzed amide allylation of N methyl isatin. Org. Lett..

[13] Irakusne L., Santiago R., Javier I., Florenci V. (2007). Highly Stereoselective Epoxidation of α-Methyl-γ-hydroxy-α,β-unsaturated Esters: Rationalization and Synthetic Applications. J. Org. Chem..

[14] Antonio G.G., Margarita H.S., Juan I.P. (2002). Synthesis and Antiproliferative Activityof a New Compound Containing an α-Methylene-γ-Lactone Group. J. Med. Chem..

[15] Romeo R., Pier G.B., Mojgan A.T., Jaime Bermejo, Francisco E., Monica B., Roberto G. (2005). Design, Synthesis, and Biological Evaluation of Hybrid Molecules Containing α-Methylene-γ-Butyrolactones and α-Bromoacryloyl Moieties. J. Med. Chem..

[16] Miyazawa M., Shimabayashi H., Hayashi S., Hashimoto S., Nakamura S., Kosaka H., Kameoka H. (2000). Synthesis and Biological Activity of α-Methylene-γ-lactones as New Aroma Chemicals. J. Agric. Food Chem..

[17] Feng J.T., Zhang Y.M., Wang J.R., Zhang X. (2007). Synthesis and antifungal activities of carabrone derivatives. Chin. J. Pestic. Sci..

[18] Feng J.T., Ma Z.Q., Wang J.R., Wang Z.H., Su Z.S., Li G.Z., Zhang X. (2009). Carabrane Type Sesquiterpene Lactone Compound with Sterilization Activity Separated from *Carpesium macrocephalum* Franch.et Sav and Its Application. China Patent.

[19] Feng J.T., Ma Z.Q., Li J., He J., Xu H., Zhang X. (2010). Synthesis and antifungal activity of carabrone derivatives. Molecules.

[20] Feng J.T., Wang H., Ren S.X., He J., Liu Y., Zhang X. (2012). Synthesis and Antifungal Activities of Carabrol Ester Derivatives. J. Agric. Food Chem..

[21] Feng J.T., Wang D.L., Wu Y.L., Yan H., Zhang X. (2013). New antifungal scaffold derived from a natural pharmacophore: Synthesis of α-methylene-γ-butyrolactone derivatives and their antifungal activity against *Colletotrichum lagenarium*. Bioorg. Med. Chem. Lett..

[22] Gao Y., Li J., Song Z., Song J., Shang S., Xiao G., Wan Z., Rao X. (2015). Turning renewable resources into value-added products: Development of rosin-based insecticide candidates. Ind. Crop. Prod..

[23] García I., Fall Y., Gómez G., González-Díaz H. (2011). First computational chemistry multi-target model for anti-Alzheimer, anti-parasitic, anti-fungi, and anti-bacterial activity of GSK-3 inhibitors *in vitro*, *in vivo*, and in different cellular lines. Mol. Divers..

[24] Prado-Prado F.J., Borges F., Perez-Montoto L.G., González-Díaz H. (2009). Multi-target spectral moment: QSAR for antifungal drugs *vs.* different fungi species. Eur. J. Med. Chem..

[25] Choudhury P.K., Foubelo F., Yus M. (1999). Direct indium-promoted preparation of α-methylene-γ-lactones from 2-(bromomethyl) acrylic acid and carbonyl compounds. Tetrahedron.

[26] Song J., Wang Z., Findlater A., Han Z., Jiang Z., Chen J., Zheng W., Hyde S. (2013). Terpenoid mosquito repellents: A combined DFT and QSAR study. Bioorg. Med. Chem. Lett..

[27] Wang Z., Song J., Chen J., Song Z., Shang S., Jiang Z., Han Z. (2008). QSAR study of mosquito repellents from terpenoid with a six-member-ring. Bioorg. Med. Chem. Lett..

[28] Liao S., Song J., Wang Z., Chen J., Fan G., Song Z., Shang S., Chen S., Wang P. (2014). Molecular interactions between terpenoid mosquito repellents and human-secreted attractants. Bioorg. Med. Chem. Lett..

[29] Li J., Gao Y., Shang S., Rao X.J., Wang Z. (2014). Synthesis and quantitative structure–activity relationship (QSAR) studies of novel rosin-based diamide insecticides. RSC Adv..

[30] Li J., Xiao G., Shang S., Rao X. (2014). A QSAR Study of the Acrylpimaric Acid Derivatives as the Inhibitors of NCI-H460. Lett. Drug Des. Discov..

[31] Karelson M., Lobanov V.S., Katritzky A.R. (1996). Quantum-Chemical Descriptors in QSAR/QSPR Studies. Chem. Rev..

[32] Thanikaivelan P., Subramanian V., Rao J.R., Nair B.U. (2000). Application of quantum chemical descriptor in quantitative structure activity and structure property relationship. Chem. Phys. Lett..

[33] Ellis G.W.L., Tavares D.F., Rauk A. (1985). The mechanism of an intramolecular Michael addition: A MNDO study. Can. J. Chem..

[34] Lavallee J.F., Berthiaume G., Deslongchamps P. (1986). Intramolecular Michael addition of cyclic β-ketoester on conjugated acetylenic ketone. Tetrahedron Lett..

[35] Kalani K., Yadav D., Khan F., Srivastava S., Suri N. (2012). Pharmacophore, QSAR, and ADME based semisynthesis and *in vitro* evaluation of ursolic acid analogs for anticancer activity. J. Mol. Model..

[36] Roy P.P., Kovarich S., Gramatica P. (2011). QSAR Model Reproducibility and Applicability: A Case Study of Rate Constants of Hydroxyl Radical Reaction Models Applied to Polybrominated Diphenyl Ethers and (Benzo-)Triazoles. J. Comput. Chem..

[37] Schmidt T.J., Nour A.M.M., Khalid S.A., Kaiser M., Brun R. (2009). Quantitative Structure-Antiprotozoal Activity Relationships of Sesquiterpene Lactones. Molecules.

[38] Heilmann J., Waseschaa M.R., Schmidt T.J. (2001). The Influence of Glutathione and Cysteine Levels on the Cytotoxicity of Helenanolide Type Sesquiterpene Lactones Against KB Cells. Bioorg. Med. Chem..

[39] Li S.K., Ji Z.Q., Zhang J.W., Guo Z.Y., Wu W.J. (2010). Synthesis of 1-Acyl-3-isopropenylbenzimidazolone Derivatives and Their Activity against *Botrytis cinerea*. J. Agric. Food Chem..

[40] Frisch M.J., Trucks G.W., Schlegel H.B., Scuseria G.E., Robb M.A., Cheeseman J.R., Montgomery J.A., Vreven T., Kudin K.N., Burant J.C. (2004). Gaussian 03.

[41] Katritsky A., Karelson M., Lobanov V.S., Dennington R., Keith T.A., Keith R.D.A.T. (2004). Codessa 2.7.15.

[42] Dewar M.J.S., Holder A.J., Roy I., Dennington D., Liotard D.A., Truhlar D.G., Keith T.A., Millam J.M., Harris C.D. (2004). AMPAC 9.3.1.

[43] Li J., Tian X., Gao Y., Shang S., Feng J., Zhang X. (2015). A value-added use of volatile turpentine: Antifungal activity and QSAR study of β-pinene derivatives against three agricultural fungi. RSC Adv..

